# Autoimmunity to stromal-derived autoantigens in rheumatoid ectopic germinal centers exacerbates arthritis and affects clinical response

**DOI:** 10.1172/JCI169754

**Published:** 2024-04-30

**Authors:** Elisa Corsiero, Mattia Caliste, Lucas Jagemann, Liliane Fossati-Jimack, Katriona Goldmann, Cankut Cubuk, Giulia M. Ghirardi, Edoardo Prediletto, Felice Rivellese, Cristiano Alessandri, Mark Hopkinson, Behzad Javaheri, Andrew A. Pitsillides, Myles J. Lewis, Costantino Pitzalis, Michele Bombardieri

**Affiliations:** 1 Centre for Experimental Medicine and Rheumatology, William Harvey Research Institute, Barts and The London School of Medicine and Dentistry, Queen Mary University of London (QMUL), London, United Kingdom.; 2 Arthritis Center, Department of Clinical, Internal Medicine, Anesthesiological and Cardiovascular Sciences, Sapienza University of Rome, Rome, Italy.; 3 Comparative Biomedical Sciences Centre, Royal Veterinary College, London, United Kingdom.; 4 IRCCS Istituto Clinico Humanitas Via Manzoni, Rozzano (Milano), Italy.

**Keywords:** Autoimmunity, Autoimmune diseases, Immunoglobulins, Rheumatology

## Abstract

Ectopic lymphoid structures (ELSs) in the rheumatoid synovial joints sustain autoreactivity against locally expressed autoantigens. We recently identified recombinant monoclonal antibodies (RA-rmAbs) derived from single, locally differentiated rheumatoid arthritis (RA) synovial B cells, which specifically recognize fibroblast-like synoviocytes (FLSs). Here, we aimed to identify the specificity of FLS-derived autoantigens fueling local autoimmunity and the functional role of anti-FLS antibodies in promoting chronic inflammation. A subset of anti-FLS RA-rmAbs reacting with a 60 kDa band from FLS extracts demonstrated specificity for HSP60 and partial cross-reactivity to other stromal autoantigens (i.e., calreticulin/vimentin) but not to citrullinated fibrinogen. Anti-FLS RA-rmAbs, but not anti–neutrophil extracellular traps rmAbs, exhibited pathogenic properties in a mouse model of collagen-induced arthritis. In patients, anti-HSP60 antibodies were preferentially detected in RA versus osteoarthritis (OA) synovial fluid. Synovial *HSPD1* and *CALR* gene expression analyzed using bulk RNA-Seq and GeoMx-DSP closely correlated with the lympho-myeloid RA pathotype, and HSP60 protein expression was predominantly observed around ELS. Moreover, we observed a significant reduction in synovial HSP60 gene expression followed B cell depletion with rituximab that was strongly associated with the treatment response. Overall, we report that synovial stromal-derived autoantigens are targeted by pathogenic autoantibodies and are associated with specific RA pathotypes, with potential value for patient stratification and as predictors of the response to B cell–depleting therapies.

## Abstract 2

A synovial autoreactive humoral immune response against synovial stroma-derived autoantigens generates autoantibodies with pathogenic capacity in rheumatoid arthritis.

## Graphical abstract

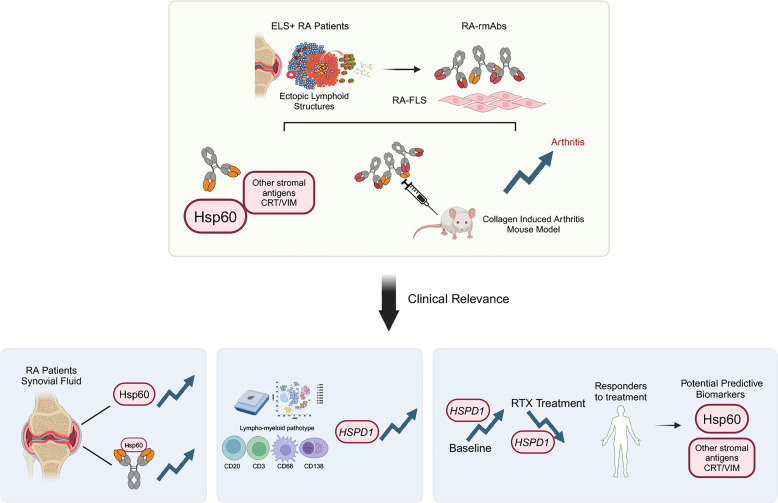

## Introduction

Rheumatoid arthritis (RA) is the most common inflammatory, erosive polyarthritis and affects approximately 0.5%–1.5% of the worldwide population. RA is characterized by breach of self-tolerance and production of several autoantibodies including anti-citrullinated protein/peptide antibodies (ACPAs) that can be manufactured within synovial ectopic lymphoid structures (ELSs), as previously described (1–3).

Fibroblast-like synoviocytes (FLSs) have been shown to play a critical role in the pathogenesis of RA contributing to the initiation, evolution, and perpetuation of the disease (4–7). Besides contributing to joint damage by secreting MMPs, RA-FLSs can also release proinflammatory cytokines and chemokines (8, 9). Previous data have demonstrated that RA-FLSs express functional TLR1–TLR6, with overexpression of TLR3 and TLR4 in synovial tissues of patients with long-standing as well as early RA (10). Notably, it has been proposed that TLR4 endogenous ligands, such as HSPs, may be required to induce proinflammatory cytokine production in RA-FLSs (11). Several mediators of the stress response, such as HSPs and calreticulin, belong to molecular chaperones that become highly expressed during inflammation, including in the RA synovium (12–14). HSPs can also interact with immune cells of both innate and adaptive arms, leading to either activation of the humoral immune response with production of anti-HSP antibodies or stimulation of immune-regulatory mechanisms (15). These proteins have a long history in arthritis research, and previous studies have identified anti-HSP60, anti-HSP70, and anti-calreticulin autoantibodies in the sera of patients with RA (16–18). HSPs can be found intracellularly, at the cell surface, and extracellularly. It has been proposed that the presentation of HSPs in the extracellular environment by stromal cells in RA may modulate the immune system and sustain an inflammatory response (19).

Previously, we have shown that a subset of RA synovial recombinant monoclonal antibodies (rmAbs) generated from single CD19^+^ synovial B cells isolated from synovial tissue of patients with ELS^+^ACPA^+^ RA are able to recognize stromal-derived autoantigens (18), prompting further investigation of other potentially stromal-derived autoantigens targeted by the B cells differentiated in the ELS^+^ synovium and their pathogenic potential in contributing to chronic inflammation. Thus, in this work, we performed a comprehensive analysis aimed at identifying key stromal-derived autoantigens fueling the local autoimmune response, their distribution and expression among different synovial RA pathotypes, and the functional effect of anti-FLS RA-rmAbs in the collagen-induced arthritis (CIA) mouse model.

## Results

### Identification of HSP60 as an antigenic target of a subset of synovial B cell–derived anti-FLS RA-rmAbs with partial cross-reactivity to other stromal-related antigens.

**Figure 1:**
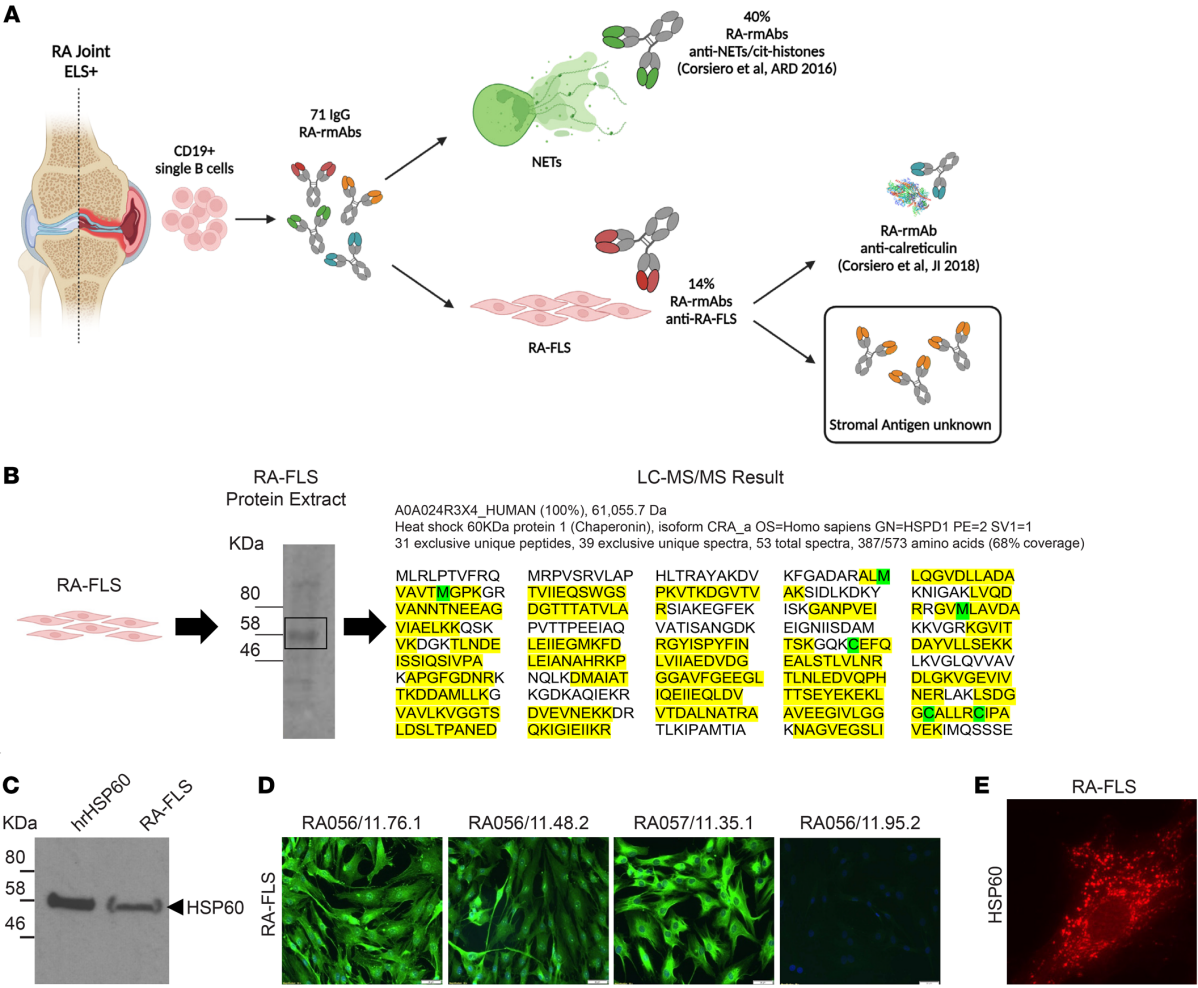
Synovial RA-rmAbs display immunoreactivity to RA-FLS. (**A**) Schematic summarizing the origin and reactivity characterization of the RA-rmAbs (*n* = 71) against NETs/cit-histones and RA-FLS. Schematic in **A** was created using BioRender.com. (**B**) RA-FLS protein extract was probed with an anti-FLS RA-rmAb. A protein of approximately 60 kDa was excised and analyzed by MS. The analysis detected a high amount of sequence coverage (68%) across the full length of the HSP60 protein in the RA-FLS protein extract. (**C**) rhHSP60 and RA-FLS protein extracts were subjected to Western blotting and probed with an anti–human HSP60 antibody. (**D**) Representative immunofluorescence images of RA-FLS incubated with the RA-rmAbs, showing immunoreactivity of 3 RA-rmAbs to FLS-derived antigens (green). RA056/11.95.2 was used as a negative control. Nuclei were stained with DAPI (blue). Original magnification, ×20. (**E**) Representative immunofluorescence image of RA-FLS showing expression of HSP60 (red). Original magnification, ×40.

Of 71 rmAbs generated from 4 ELS^+^ RA synovial tissues, we previously showed that 10 RA-rmAbs display anti-FLS immunoreactivity with variable immunofluorescence patterns (Figure 1A and Supplemental Figure 1; supplemental material available online with this article; https://doi.org/10.1172/JCI169754DS1). Liquid chromatography tandem mass spectrometry (LC-MS/MS) analysis of a 60 kDa band of total protein detected via Western blotting by several RA-rmAbs targeting RA-FLSs identified HSP60 as one of the most abundant proteins (among >100 detected by the LC-MS/MS analysis), with a high amount of sequence coverage (68%) across the full length of HSP60 (Figure 1B). HSP60 is a molecular chaperone expressed in different intracellular compartments (e.g., cytosol) as well as extracellularly that migrates to an approximately 58 kDa position in SDS-PAGE (Figure 1C).

Therefore, we next performed immunofluorescence studies to determine whether HSP60 and/or other stromal-related antigens were the target of the subset of RA-rmAbs reactive to RA-FLSs (Figure 1D and Supplemental Figure 1). We confirmed the expression of HSP60 in RA-FLSs by Western blotting of the RA-FLS total protein extract and by immunofluorescence staining (Figure 1, C and E).

**Figure 2:**
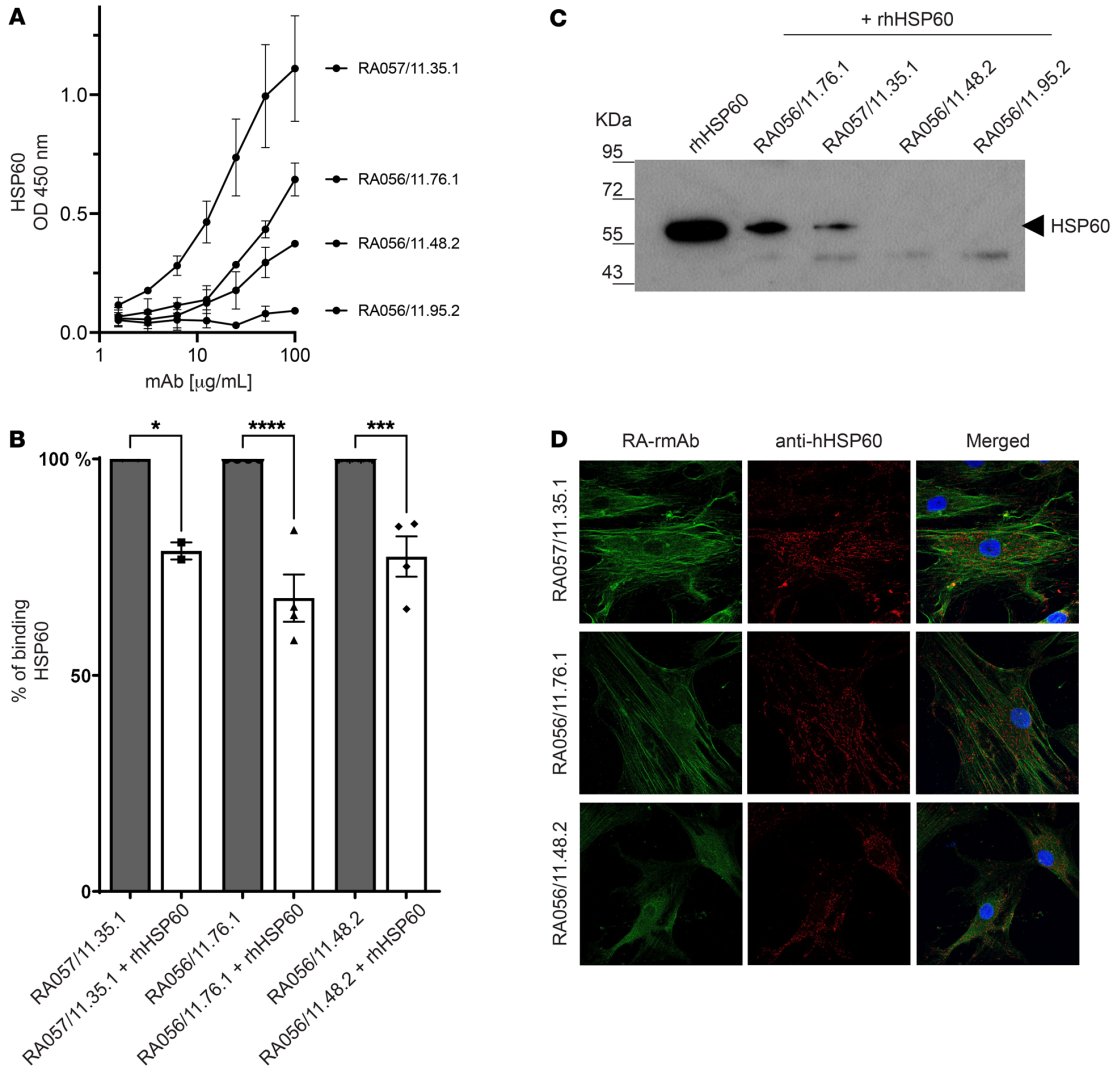
RA-rmAbs with anti-FLS reactivity recognize HSP60. (**A**) Binding of RA057/11.35.1, RA056/11.76.1, and RA056/11.48.2 rmAbs and a negative RA-rmAb (RA056/11/95.2) to rhHSP60. All RA-rmAbs were tested at a concentration of 100 mg/mL followed by 6 serial dilutions (1:2). (**B**) Competitive binding of the 3 RA-rmAbs to rhHSP60 preincubated with or without soluble HSP60 (competitor). Results are expressed as a percentage of HSP60 binding. (**C**) IP results of rhHSP60 and the RA-rmAbs. As a control, rhHSP60 alone was loaded. (**D**) Representative immunofluorescence image showing staining for HSP60 (red) and RA-rmAbs (green). Nuclei were stained with DAPI (blue). Original magnification, ×63. The data are the results of 3 independent experiments. *P < 0.05, ***P < 0.001, and ****P < 0.0001, by 1-way ANOVA with multiple comparisons.

We then quantitatively assessed in an ELISA the binding of the anti-FLS RA-rmAbs to a recombinant human HSP60 (rhHSP60) protein. We identified 3 RA-rmAbs (RA057/11.35.1, RA056/11.76.1, RA056/11.48.2) that showed binding to the rhHSP60 in a dose-dependent manner (Figure 2A). The RA057/11.35.1 rmAb showed the strongest reactivity to rhHSP60. In contrast, 1 RA-rmAb, RA056/11.95.2, which did not bind to RA-FLS (Figure 1D) and was thus used as a negative control, failed to show any binding to rhHSP60 (Figure 2A). The observed immunoreactivity against HSP60 was not due to polyreactivity, as none of the 3 RA-rmAbs displayed polyreactivity to multiple structurally unrelated antigens (Supplemental Figure 2) (2, 20, 21). We also investigated whether the 3 RA-rmAbs with anti-HSP60 immunoreactivity displayed enhanced binding to an in vitro citrullinated form of HSP60 (cit-rHSP60) (Supplemental Figure 3A). As shown in Supplemental Figure 3B, only RA056/11.76.1 rmAbs displayed increased binding to cit-rHSP60. Next, the 3 RA-rmAbs were tested for their reactivity in ELISAs to other RA-associated antigens including anti–cyclic citrullinated peptide (anti-CCP) ELISA, citrullinated fibrinogen, and the FLS-associated antigens calreticulin and vimentin, which are known RA autoantigens and migrate to a similar 60 kDa position in RA-FLS protein extract electrophoresis. As shown in Supplemental Figure 4, we also observed a variable degree of reactivity of the anti-HSP60 RA-rmAb to calreticulin and vimentin, whereas only anti-RA056/11.76.1 rmAb displayed low citrullinated fibrinogen–binding activity. We next conducted a competitive binding assay to further confirm whether HSP60 was recognized by the RA-rmAbs. As shown in Figure 2B, incubation of the RA-rmAbs with soluble rhHSP60 significantly reduced, but did not completely abrogate, the binding to HSP60 in the ELISA. In order to support the HSP60/RA-rmAb binding results, IP assays were performed. As shown in Figure 2C, IP of rhHSP60 with 2 RA-rmAbs (RA057/11.35.1 and RA056/11.76.1) showed a band of approximately 60 kDa, confirming the ELISA data. The absence of signal for the RA-rmAb RA057/11.48.2 might be explained by the low level of binding to HSP60 observed in the ELISA.

We next sought to confirm that the RA-rmAbs target FLS-derived HSP60 by immunofluorescence staining. As shown in Figure 2D, double-immunofluorescence staining with the 3 RA-rmAbs in combination with an anti–human HSP60 antibody demonstrated a partial cellular colocalization with the HSP60. We quantified the degree of colocalization using ImageJ (NIH), with a Pearson’s correlation coefficient of *r* = 0.4–0.5, which suggested a positive correlation (22, 23). Since the staining morphology of our anti-FLS rmAbs suggested partial binding to cytoskeletal proteins, either through direct biding or interaction with HSP60 (24), we performed double-immunofluorescence staining of the RA-rmAbs with an anti–human F-actin antibody showing variable but largely incomplete colocalization for the 3 RA-rmAbs with a structural FLS antigen (Supplemental Figure 5).

### Anti-FLS antibodies accelerate arthritis in the CIA mouse model.

**Figure 3:**
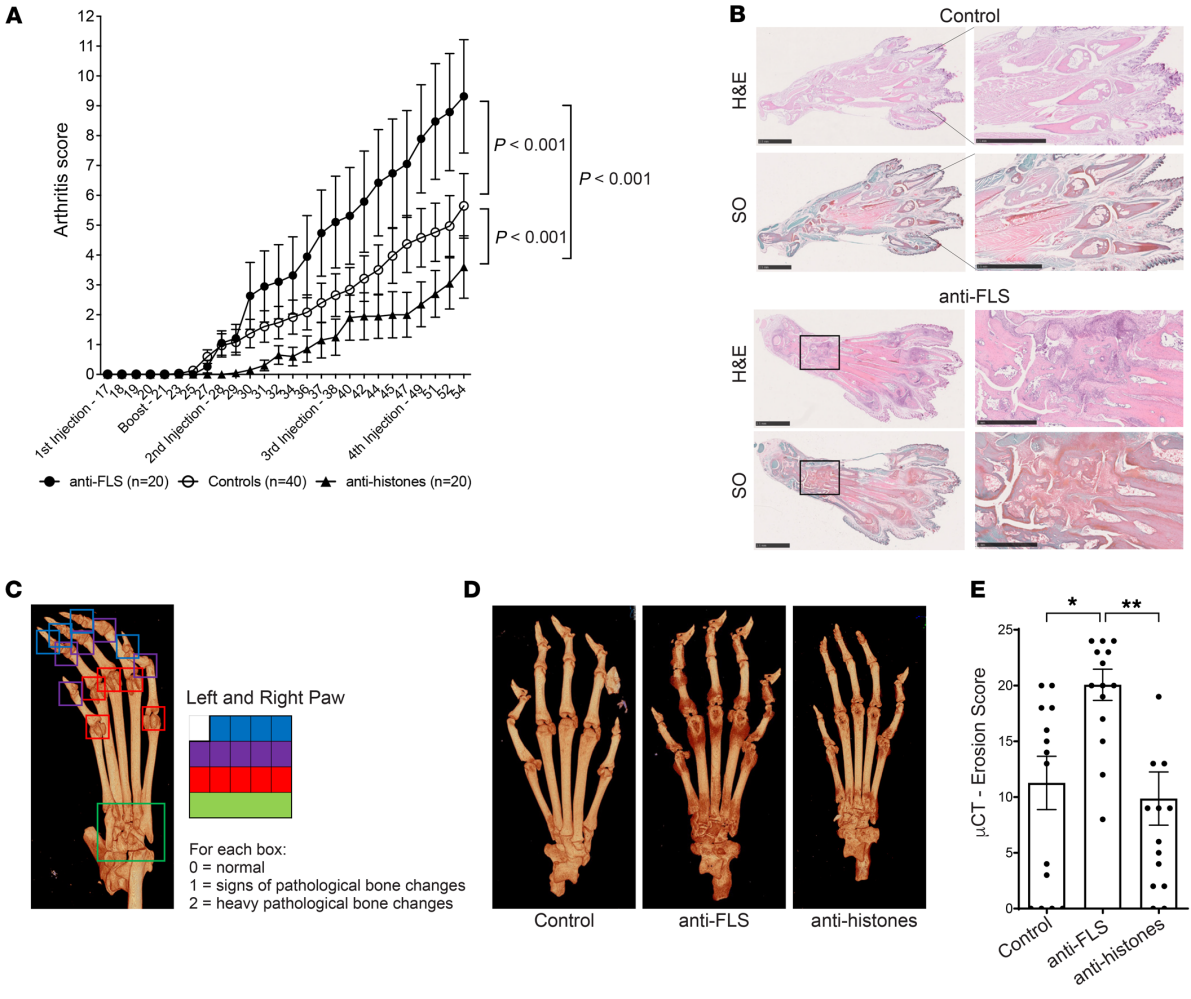
Effect of anti-FLS RA-rmAbs in CIA. (**A**) Graph shows the arthritis scores for CIA DBA/1 mice treated i.p. with anti-FLS rmAbs (*n* = 20), PBS (controls, *n* = 40), and anti-histone rmAbs (*n* = 20). Anti-FLS rmAbs were administered on days 17, 28, 38, and 49. The anti-FLS rmAbs cocktail consisted of RA056/11.76.1, RA057/11.35.1, and RA057/11.89.1 antibodies. The anti-histone rmAbs cocktail consisted of RA015/11.58, RA015/11.88, and RA015/11.91 antibodies. Results are from 2 independent experiments. The scores are reported as the mean of the sum of the score ± SEM assessed for each paw of the mouse. The *P* values correspond to the treatment × time element from the ANOVA linear model between groups. (**B**) Representative IHC (top) and safranin-O (SO) (bottom) images of control- and anti-FLS–treated mice. Scale bars: 2.5 mm. Original magnification, ×1.5 (enlarged insets, control) and ×23 (anti-FLS). (**C**) Representative μCT scan of a hind paw from a CIA mouse and the scoring system used to evaluate bone erosion (erosion score). The score was given for each digit (color-coded area), with a maximum score of 24. 0 = normal; 1 = signs of pathological bone changes; 2 = heavy pathological bone changes. (**D**) Representative μCT scan images of control-treated, anti-FLS–treated (*n* = 5) and anti-histone–treated (*n* = 5) mice. (**E**) Graph shows the μCT erosion score for CIA DBA/1 mice treated with PBS, anti-FLS RA-rmAbs, or anti-histone RA-rmAbs. Five representative hind paws were used for each group. The scoring was done blindly by 3 independent evaluators and is reported as the mean of the sum ± SEM. *P < 0.05 and **P < 0.01, by 1-way ANOVA with multiple comparisons.

We explored whether a cocktail of anti-FLS antibodies containing the RA057/11.35.1 and RA056/11.76.1 anti-HSP60 antibodies could have a functional role in the CIA mouse model. CIA was induced by intradermal injection of 2 mg/mL bovine collagen type II on day 0, followed by a boost on day 21. Anti-FLS RA-rmAbs were administered i.p. at 4 time points. As a control, we used both PBS and a mix of RA-rmAbs targeting histone proteins, which were used as a negative control (2, 25). We found that the anti-FLS RA-rmAbs were able to enhance the arthritis score and increase inflammation in the paws compared with the control-treated group (Figure 3A). In contrast, the mice treated with anti-histone RA-rmAbs (2) showed a reduction in the arthritis score (Figure 3A), similar to previous data (25). Histological evaluation of the hind paws showed a large influx of inflammatory cells and signs of cartilage damage in the anti-FLS RA-rmAbs group versus the PBS- and anti-histone RA-rmAb–treated groups (Figure 3B and Supplemental Figure 6). Moreover, micro-CT (μCT) analysis confirmed the increased bone erosion induced by the anti-FLS RA-rmAbs compared with both the control- and anti–histone RA-rmAb–treated group (Figure 3, C–E).

### Antigenic targets of anti-FLS rmAbs such as HSP60 are preferentially expressed in the lympho-myeloid pathotype, colocalize with synovial ELSs, and are upregulated in FLSs derived from leukocyte-rich RA joints.

**Figure 4:**
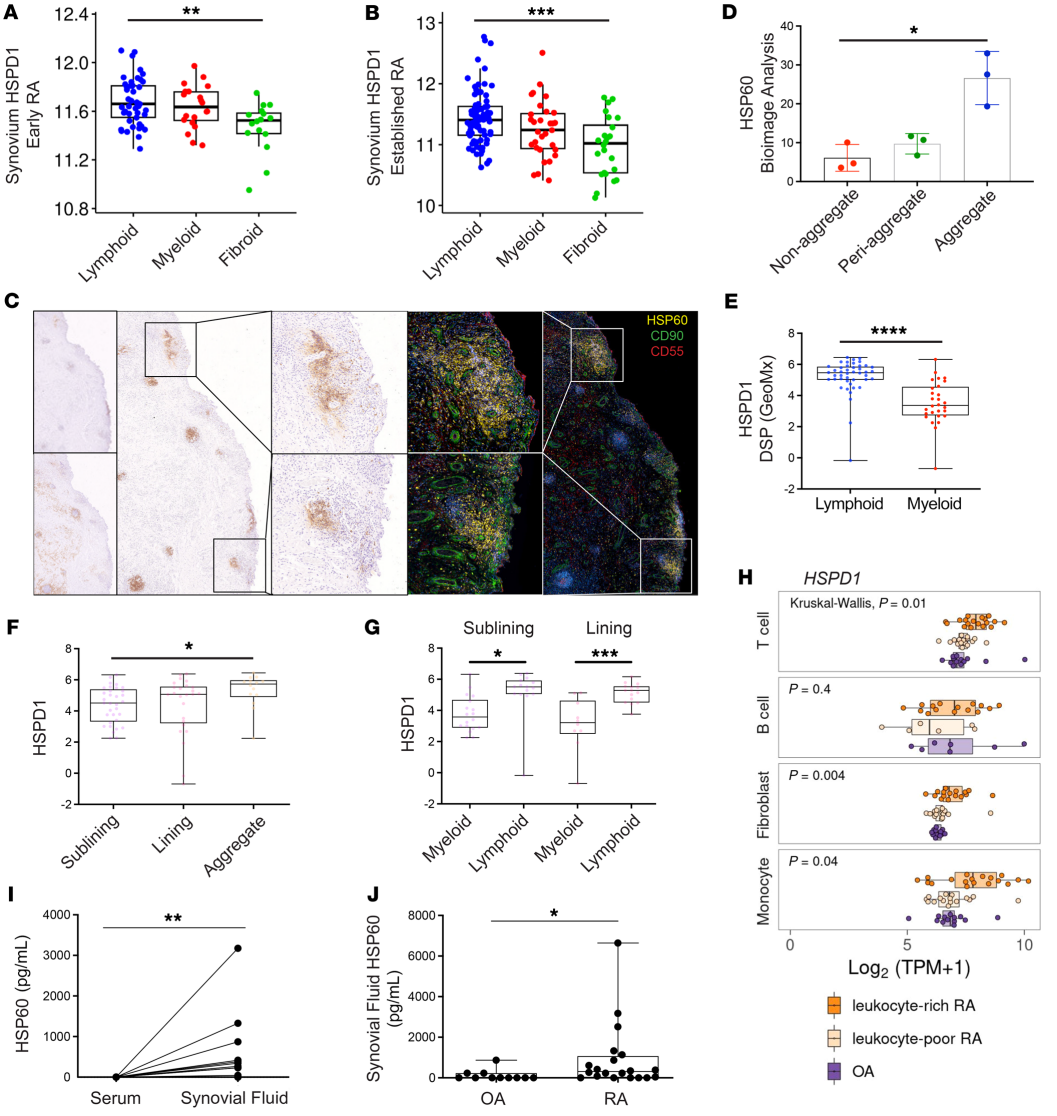
HSP60 expression in synovial tissue and SF of patients with RA. Synovium *HSPD1* gene evaluation compared across histological pathotype in patients with early (**A**) and established (**B**) RA. Unit *y* axis: regularized log-normalized read. (**C**) Representative immunofluorescence and IHC images of synovial tissue from patients with RA showing staining for HSP60 (yellow)/CD90^+^ (green)/CD55^+^ (red) synovial fibroblasts and for CD20 (B cells), CD3 (T cells), and CD138 (plasma cells). Nuclei were stained with DAPI (blue). Original magnification, ×2 and ×10 (enlarged insets). (**D**) Quantification of HSP60 differential expression in RA synovial tissue (RA patients, *n* = 3) by QuPath analysis. Statistical analysis was done by Friedman’s test. (**E**) *HSPD1* was differentially expressed in the lympho-myeloid (lymphoid) versus diffuse-myeloid (myeloid) pathotypes using GeoMx DSP data. (**F**) *HSPD1* differential expression in the sublining, lining, and aggregated area using GeoMx DSP data. In **E** and **F**, statistical analysis was performed using the Mann-Whitney *U* test. (**G**) *HSPD1* differential expression in the sublining and lining in diffuse-myeloid (myeloid) versus lympho-myeloid (lymphoid) pathotypes. Scatterplot shows individual regions of interest (ROIs)/. Statistical analysis was done using the Kruskal-Wallis test. (**H**) scRNA-Seq profiling of *HSPD1* in synovial fibroblasts in leukocyte-rich/leukocyte-poor RA and OA patients (https://immunogenomics.io/ampra/). (**I**) Graph shows HSP60 levels (pg/mL) in matched sera and SF from patients with RA (*n* = 14). Statistical analysis was done with the Wilcoxon test. (**J**) Box plot displays SF HSP60 levels in RA (*n* = 20) versus OA (*n* = 11) patients. Statistical analysis was done using the Mann-Whitney *U* test. Each data point represents an individual patient. *P < 0.05, **P < 0.01, ***P < 0.001, and ****P < 0.0001.

The distribution of HSP60 within RA synovial tissue and in matched synovial fluid (SF) and serum samples from patients with RA was examined by bulk RNA-Seq, immunofluorescence staining, and ELISA, respectively. RNA-Seq expression for *HSPD1* (gene name for HSP60) within synovial tissue was compared between the lympho-myeloid, diffuse-myeloid, and pauci-immune fibroid histological groups in patients with early or established RA using bulk RNA-Seq data generated by us from synovial biopsies obtained as part of the Pathobiology of Early Arthritis Cohort (PEAC) study and the R4RA randomized clinical trial (26–28). *HSPD1* was elevated in the lympho-myeloid versus the pauci-immune fibroid and diffuse-myeloid in both early (*P* = 0.0012) and established RA (*P* = 0.0015) patients (Figure 4, A and B). Moreover, at the protein level, HSP60 showed a preferential distribution around lymphocyte aggregates and periaggregates within the RA synovial tissue (Figure 4, C and D). In addition, we reanalyzed GeoMx Digital Spatial Profiler (DSP) data from the R4RA trial to characterize the spatial positioning of the *HSPD1* transcript in association with the different RA histopathotypes (27). Consistent with the bulk RNA-Seq data, *HSPD1* expression was significantly higher in the lympho-myeloid group versus the diffuse-myeloid group (*P* < 0.0001; Figure 4E). Like the HSP60 distribution within the RA synovial tissue determined by immunofluorescence, the spatial positioning of *HSPD1* was observed mainly around lymphocyte aggregates independently of the sublining and lining areas (Figure 4, F and G). Moreover, we detected similar levels of HSP60 protein expression in the synovial sublining and lining fibroblasts, with HSP60 protein preferentially observed around lymphocyte aggregates, as previously mentioned (Figure 4C). This observation was supported by scRNA-Seq data from the Accelerating Medicines Partnership (AMP) RA database, in which *HSPD1* was found to be highly expressed in fibroblasts from the patients with leukocyte-rich RA (29) (Figure 4H). However, *HSPD1* expression was also detected in other cell types, like monocytes (Figure 4H). Finally, we observed higher levels of HSP60 protein in the SF of patients with RA compared with HSP60 levels at the systemic level (Figure 4I). Accumulation of HSP60 in the SF was preferentially observed in patients with RA (*n* = 20) versus patients with osteoarthritis (OA) (*n* = 11) (Figure 4J).

Similar to HSP60, calreticulin (*CALR*) gene expression was significantly higher in the lympho-myeloid pathotype in bulk synovial RNA-Seq results and in spatial association with lymphoid aggregates using GeoMx analysis of patients with established RA (Supplemental Figure 7, A–D), supporting the conclusion that stromal-derived autoantigens involved in the cellular stress response were upregulated in RA patients with the lympho-myeloid pathotype and contributed to the generation of pathogenic autoantibodies.

### Anti-HSP60 antibodies are elevated in the SF of patients with RA.

**Figure 5:**
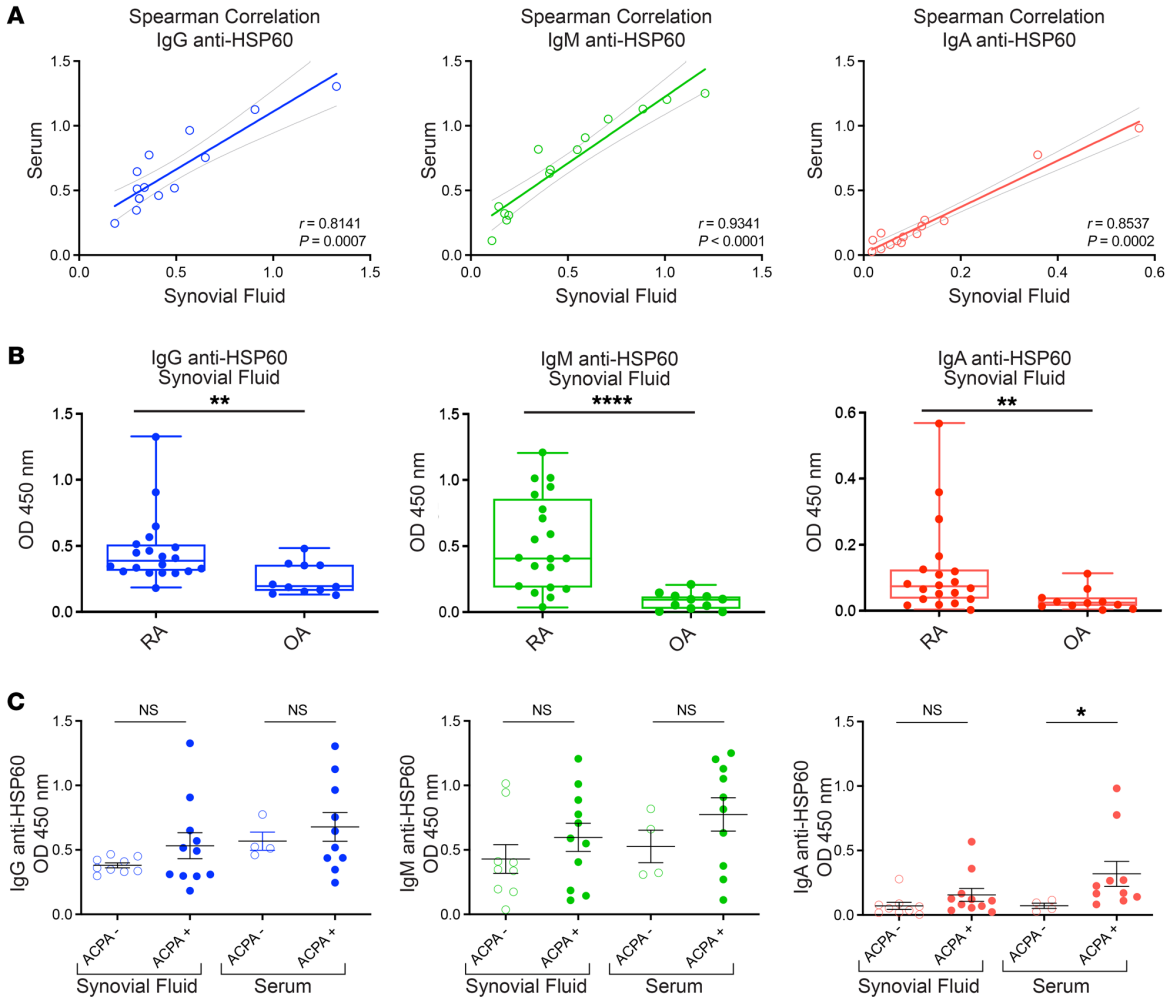
Expression of anti-HSP60 antibodies in serum and SF of patients with RA or OA. (**A**) Graphs show the correlation of matching RA SF IgG, IgM, and IgA binding to HSP60 with RA sera IgG, IgM, and IgA binding to HSP60. (**B**) Box plots display the levels of IgG, IgM, and IgA anti-HSP60 antibodies in SF of RA (*n* = 20) versus OA (*n* = 11) patients. (**C**) Scattered dot plots show IgG, IgM, and IgA binding to HSP60 in SF and sera of ACPA^+^ versus ACPA^–^ RA patients. For **B** and **C**, the results are expressed as absorbance at 450 nm, and each data point represents an individual patient. *P < 0.05, **P < 0.01, and ****P < 0.0001, by Mann-Whitney *U* test.

We then investigated the prevalence of anti-HSP60 antibodies in matched SF and serum samples from patients with RA. Serum and SF anti-HSP60 antibodies were measured by ELISA using rhHSP60. As shown in Figure 5A, binding of IgG, IgM, and IgA to HSP60 in SF was positively correlated to RA serum levels. We detected a significant difference of SF IgG, IgM, and IgA anti-HSP60 antibodies between patients with OA or RA, with an increase of IgM levels observed in the SF of patients with RA (Figure 5B and Supplemental Figure 8, A and B). Only 10% of the RA SF tested did not have anti-HSP60 antibodies. Ten percent of RA SF was characterized by the presence of IgG, IgM, and IgA binding to HSP60 (triple-positive), with 25% double-positive and 55% single-positive binding (Supplemental Figure 8C). Finally, patients with RA were divided into ACPA^+^ (serum, *n* = 11; SF, *n* = 10) and ACPA^–^ (serum, *n* = 9; SF, *n* = 4) groups using a conventional anti-CCP2 test. As shown in Figure 5C, the levels of antibodies against HSP60 in ACPA^+^ RA patients were significantly increased for IgA antibodies, with only a trend for IgG and IgM anti-HSP60 antibodies for both serum and SF samples.

### Synovial HSP60 expression is predictive of the clinical response to rituximab and is modulated by B cell depletion in good clinical responders.

**Figure 6:**
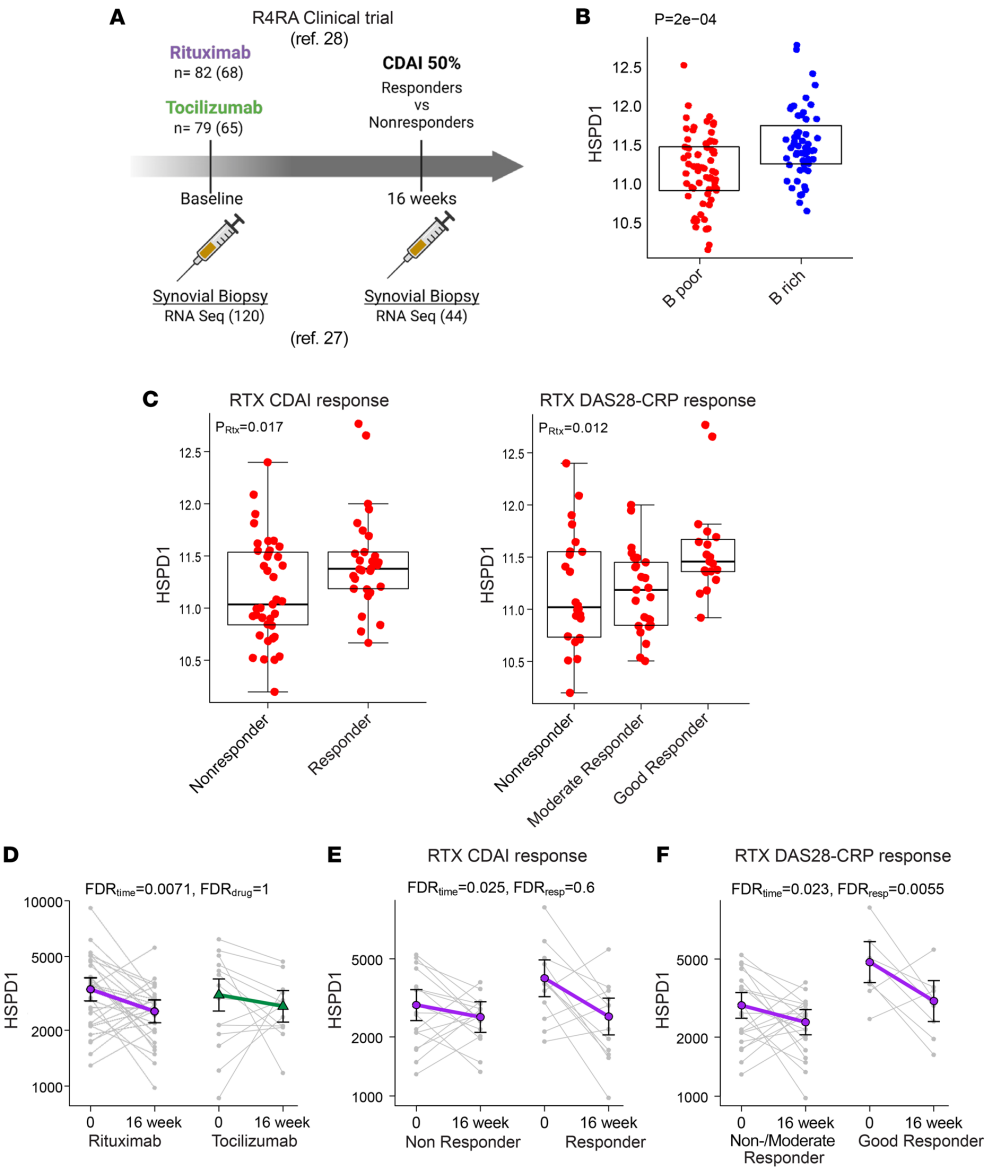
HSP60 analysis of paired pre- and post-rituximab treatment synovial biopsies. (**A**) Schematic representation of the R4RA clinical trial (27, 28). At baseline, patients with RA underwent a synovial biopsy of a clinically active joint for histological evaluation. Following the synovial biopsy, patients were randomized 1:1 to either the rituximab or tocilizumab treatment group. An optional repeated synovial biopsy of the same joint previously sampled was performed at 16  weeks, followed by histological and transcriptomic evaluation. A CDAI of 50% was used as a primary endpoint to define responders and nonresponders to treatment at 16 weeks. Patients were initially followed up every 4 weeks, with the end of the trial at 48 weeks. Numbers in parentheses represent patients with available RNA-Seq data. (**B**) Synovium *HSPD1* variance-stabilizing transformation (VST) gene expression in patients stratified by histology into B cell–poor (B poor) and B cell–rich (B rich) categories. (**C**) *HSPD1*-normalized synovial gene expression levels assessed at baseline in CDAI nonresponders versus responders and DAS28-CRP moderate/nonresponders versus responders to rituximab treatment. (**D**) *HSPD1* RNA-Seq counts were assessed at baseline and 16 weeks following rituximab and tocilizumab treatment in paired synovial biopsies (rituximab, *n* = 29 individuals, *n* = 58 samples; tocilizumab, *n* = 15 individuals, *n* = 30 samples). (**E** and **F**) *HSPD1* counts were assessed at baseline and 16 weeks following rituximab treatment in paired synovial biopsies. In **D**–**F**, gray data points represent RNA-Seq counts between paired samples for individuals. Statistical analysis was performed using a negative binomial mixed-effects model on RNA-Seq counts. Overlaid green/purple data points show estimated marginal means of the fitted mixed-effects model, with error bars showing 95% CIs for the fixed effects of the mixed-effects model. To assess the clinical response, the following parameters were used: CDAI 50% improvement and DAS28-CRP European Alliance of Associations for Rheumatology (EULAR) response. RTX, rituximab.

Given the high correlation of HSP60 with the presence of ELSs in the RA joints, we assessed the longitudinal effect of rituximab therapy on synovial *HSPD1* expression before and after B cell depletion in the R4RA biopsy-driven randomized trial (Figure 6A) (27, 28). Baseline *HSPD1* gene expression was significantly higher in the B cell–rich subset of patients with RA and was associated with good clinical responders, as defined by the R4RA trial’s clinical primary endpoint, the Clinical Disease Activity Index (CDAI), and by the secondary endpoint, the Disease Activity Score 28–C-reactive protein (DAS28-CRP) (Figure 6, B and C). Hence, we next explored the longitudinal effect of rituximab on *HSPD1* within synovial tissue before and after B cell depletion. Using mixed-effects models applied to paired synovial samples (*n* = 29 individuals, *n* = 58 samples), we first observed a significant downregulation of *HSPD1* expression 16 weeks after treatment with rituximab, but not following tocilizumab therapy (Figure 6D). For tocilizumab, baseline *HSPD1* gene expression was not associated with good clinical responders, as defined by the R4RA trial clinical primary endpoint CDAI and by the secondary endpoint DAS28-CRP (Supplemental Figure 9). Interestingly, a significant downregulation of synovial *HSPD1* expression upon B cell depletion was selectively observed in good responders compared with nonresponders and moderate responders (Figure 6, E and F) using mixed-effects models applied to paired synovial samples only, with similar results if all samples were included in the models (Supplemental Figure 10A). We observed similar results also for *CALR* expression (Supplemental Figure 7, E–G), suggesting a common gene expression profile linked to cellular stress response mechanisms in RA-FLS with local release of autoantigens.

Finally, in the R4RA cohort we observed a positive correlation between serum anti-HSP60 IgG with CDAI, tender joint count, and DAS28-ESR at baseline (Supplemental Figure 10B), even though IgG anti-HSP60 antibody levels did not change over time after rituximab treatment (Supplemental Figure 11).

## Discussion

FLS or synovial fibroblasts are the main cell type of the synovial intima (or inner layer) of the synovial membrane. In RA, the synovial lining becomes hyperplastic, with a high number of activated FLSs that participate in the formation of the synovial pannus and to joint destruction via the production of proinflammatory cytokines and MMPs. In previous work, by screening a large number of rmAbs derived from locally differentiated B cells from RA ELS^+^ synovium (18), we identified a subset of antibodies specifically targeting RA-FLSs, suggesting that FLSs might act as a cellular source of autoantigens able to drive the humoral autoimmune response in RA synovial tissue. Although we identified calreticulin as a potential stromal-derived antigen, this autoantigen was recognized by only 1 synovial B cell clone diversified within the RA synovial ELSs, and as such, the antigenic target of the other anti-FLS antibodies remained unknown. Therefore, in this work, we explored other stromal-derived antigens identified by MS analysis that are potentially capable of fueling the local autoimmune response and identified HSP60 as one of the key FLS-derived autoantigens recognized by anti-FLS RA-rmAbs. HSPs are highly conserved proteins among species, and aside from their role as chaperone proteins into the cells, they have been identified as strong activators of the immune system when released in the extracellular environment (19, 30). In autoimmune diseases, particularly in RA, the humoral autoimmune response to HSP60 has been previously reported at the serum level (31, 32). HSP60 is also known to be expressed in the synovial membrane of healthy individuals as well as in patients with RA or OA, in whom it may play a protective role in stressed cells (19, 33). Stress factors include cytokines, ROS, heat, and hypoxia that can elicit the secretion of HSPs by local cells such as FLSs (19). Overexpression of HSP60 in the stressed joint can alter its localization and be released by stromal cells, which might explain an autoimmune response against this antigen (19). Autoantibodies against HSP60 have been described in sera from patients with RA, but their role is still not well understood, since they were reported to be either arthritogenic or protective in conflicting studies (19, 34).

Here, we first screened the anti-FLS RA-rmAbs for their reactivity to rhHSP60 in ELISA. We observed 3 RA-rmAbs (RA057/11.35.1, RA056/11.76.1, RA056/11.48.2) that were reactive to HSP60. The specific reactivity was confirmed by using at least 3 methods: (a) ELISA using rhHSP60 with competitive binding assays; (b) IP using rhHSP60 and the RA-rmAbs; and (c) colocalization with a commercial anti-HSP60 antibody in confocal microscopy. Confocal microscopy showed only partial colocalization, suggesting that the RA-rmAbs could recognize HSP60 also when released from the FLS in the extracellular environment. Moreover, together with the partial inhibition by preabsorption with HSP60 in the ELISA, we found that these RA-rmAbs had some level of cross-reactivity to other RA-associated antigens like calreticulin and vimentin, which are other known autoantigens in RA and migrate to a similar position in FLS protein extract electrophoresis. However, the RA-rmAbs showed no evidence of polyreactivity to structurally unrelated proteins such as dsDNA, LPS, and insulin, which are commonly used to define polyreactive autoantibodies derived from single B cells (2, 20, 21).

HSP60 has an isoelectric point of 5.7 and is characterized by negatively charged amino acids on 14% of the full sequence (35). The RA057/11.35.1 and RA056/11.76.1 rmAbs are characterized by the presence of positively charged amino acids in the complementarity-determining region 3 (CDR3) of both the heavy and light chains (Supplemental Table 1). This suggests that the interaction with HSP60 might rely on the presence of positively charged amino acids that could form a charge-charge interaction with the antigen. The RA056/11.48.2 rmAb does not have positively charged amino acids in the CDR3 of the light chain, which might explain the lower or no interaction with HSP60 observed in the ELISA and IP studies, respectively.

The biological significance and the pathological roles of anti-FLS RA-rmAbs, including the ones reactive to HSP60, has not been fully investigated as yet. Therefore, we took advantage of the unique tools that we generated (RA-rmAbs with HSP60 immunoreactivity) to examine the potential pathogenicity of anti-FLS antibodies. A cocktail of anti-FLS RA-rmAbs, including both the RA057/11.35.1 and RA056/11.76.1, was injected into the CIA mice. We observed that anti-FLS antibodies were able to exacerbate arthritis and increase joint damage compared with the PBS control and a mix of RA-rmAbs previously characterized for their reactivity to histones (2). Interestingly, the anti-histone RA-rmAbs were shown to improve the arthritis score in CIA mice compared with controls, an observation in line with previous work in which anti–cit-histone antibodies were shown to ameliorate joint inflammation in the CIA model, putatively by shielding the cit-histones presented extracellularly during inflammation and neutrophil extracellular traps (NETosis) from the immune system, thus preventing inflammation (25).

Previous studies did not detect higher levels of HSP60 in the sera of patients with RA compared with healthy donors, thus proposing that extracellular HSP60 may be generated at the site of inflammation during disease progression (36). Therefore, we first assessed the expression of HSP60 in synovial tissue of patients with RA both at the mRNA (*HSPD1*) and protein levels. Using bulk RNA-Seq data on patients with early RA and those with established RA and GeoMx DSP data from the R4RA trial, we observed that *HSPD1* (as well as *CALR*) was preferentially expressed in the lympho-myeloid pathotype and in spatial association with ELSs. Similarly, in the RA synovial tissue, we observed that HSP60 IF staining selectively accumulated around and within lymphocyte aggregates, supporting the role of autoantigens released by FLSs during cellular stress responses in fueling the local autoimmune response in the RA ELS^+^ synovium and contributing to the generation of pathogenic autoantibodies within the RA joints.

Higher levels of HSP60 were also observed in SF from patients with RA but not in the circulation. On the contrary, we could not detect HSP60 in the SF of patients with OA. The elevated levels of HSP60 in the RA joints indicated a local, but not systemic, proinflammatory role of HSP60 in the RA synovitis.

To confirm the results found at the single synovial B cell clonal level with the local production of autoantibodies in patients with RA, we assessed the humoral autoimmune response to HSP60 in the SF of patients with RA. In comparison with OA SF, IgG, IgM, and IgA autoantibodies against HSP60 were markedly increased in the SF of patients with RA. Interestingly, both RA057/11.35.1 and RA056/11.48.2 were derived from IgM^+^CD19^+^ cells, with a variable degree of somatic hypermutation in the variable heavy and/or light chain, while RA056/11.76.1 was from an IgG^+^CD19^+^ clone (Supplemental Table 2). This observation is in line with the increased SF IgM immunoreactivity to HSP60 compared with SF IgG immunoreactivity that we observed in this study.

Moreover, all isotype anti-HSP60 antibody levels were higher in ACPA^+^ patients in both the SF and at the systemic level, but only IgA isotype levels reached statistical significance. Thus, HSP60 appeared to act as an autoantigen locally released in the RA synovial tissue that sustained local humoral immunity.

Using data from the R4RA randomized trial, we were able to define the role of synovial FLS–derived autoantigen expression as a potential biomarker of a response to either rituximab or tocilizumab treatment. Interestingly, both *HSPD1* and *CALR* retained their association with the lympho-myeloid pathotype and displayed higher gene expression levels at baseline in clinical responders to rituximab (but not tocilizumab), as assessed using both CDAI and DAS28 response criteria. Similarly, in a longitudinal pre- and post-therapy analysis, synovial *HSPD1* expression was downmodulated selectively in patients with RA who had a good clinical response to rituximab, suggesting that the HSP60 autoimmunity might influence the clinical response to B cell–depleting therapy. When we investigated longitudinal changes in circulating anti-HSP60 IgG levels in the serum of patients in the R4RA trial at baseline and 16 weeks after rituximab treatment, we did not observe any significant association with the clinical response. A likely explanation is that unlike synovial autoantigen expression, circulating autoantibodies reflect not only local release from the synovium but also systemic production from secondary lymphoid organs and/or long-lived plasma cells in the bone marrow.

In summary, we identified a subset of synovial B cell clones diversified within RA synovial ELSs with anti-HSP60 immunoreactivity. These results, linked with the observation of HSP60 expression around lymphocytes aggregated in RA synovial tissue and higher levels of this protein in RA SF, suggest that HSP60 acts as a locally released autoantigen that can be targeted by autoreactive B cells. Moreover, these antibodies displayed variable cross-reactivity to other RA-FLS–associated chaperonins such as calreticulin, suggesting that several stromal autoantigens can contribute to fueling the local autoimmune response (37, 38). Notably, anti-FLS antibodies, including the RA-rmAbs targeting HSP60, appear capable of contributing to synovial inflammation and joint damage in vivo. Although more studies are needed to clarify the potential pathogenic role for anti-HSP60 autoantibodies in enhancing inflammatory reactions induced by extracellular HSPs, the identification of immunodominant epitopes recognized by anti-FLS RA-rmAbs may pave the way for the development of innovative therapeutic approaches encompassing vaccination and tolerogenic strategies.

## Methods

Details on the methods used in this study are reported in the supplemental material.

### Sex as a biological variable.

Details on the sex of the recruited participants in the PEAC and R4RA studies have been extensively reported in the original manuscripts describing the clinical and transcriptomic profiling of these cohorts (26–28, 39). The sex of the RA and OA patients in whom autoantibodies were tested in serum and/or SF is reported in Table 1. The female to male ratio was what would be expected from an RA population. Statistical analysis did not consider sex as a variable.

For experimental arthritis, only male mice were used because the susceptibility to the development of arthritis is higher in male mice than in female mice (40), which is in line with the 3R (replacement, reduction, and refinement) principles of animal research.

### Patients.

Patients with RA were diagnosed according to the revised American College of Rheumatology criteria (Table 1) (41). OA serum and SF samples were obtained in-house.

### Generation of RA-rmAbs from ELS^+^ RA synovial tissue.

RA-rmAbs were generated from single synovial CD19^+^ B cells (synovial tissue, *n* = 4), as previously reported (2, 18).

### Isolation of FLSs from RA joints.

FLSs were obtained from synovial tissue, as previously described (5). Detailed methodology is reported in the Supplemental Methods.

### ELISA for HSP60 and anti-HSP60.

HSP60 was measured in serum and SF using a commercial ELISA kit (Invitrogen, Thermo Fisher Scientific), following the manufacturer’s instructions. For anti-HSP60 antibodies, Nunc MaxiSorp ELISA plates were coated overnight with rhHSP60/cit-HSP60 (Abcam, ab113192) protein in 1× PBS at 10 μg/mL. RA-rmAbs were transferred onto an ELISA plate and incubated for 2 hours at room temperature (RT). Unbound samples were removed before incubation for 1 hour with HRP-coupled goat anti–human IgG (Bethyl Laboratories, 109-035-003). All the RA-rmAbs and controls were tested at 100 mg/mL followed by a 1:2 dilution or at 10 mg/mL for cit-HSP60. For serum and SF evaluation, anti-HSP60 Igs were measured using isotype-specific HRP-coupled anti-human antibodies (Bethyl Laboratories). For the competition assay, ELISA plates were coated overnight with rhHSP60 at 10 μg/mL in 1× PBS at 4°C. After washing and blocking with 1% BSA-1X PBS (blocking buffer) for 1 hour at RT, 50 μg/mL of selected RA-rmAbs with or without 10 μg/mL rhHSP60 in blocking buffer were added to each well in duplicate for 2 hours at RT on a shaker. Unbound samples were removed before incubation for 1 hour with HRP-coupled goat anti–human IgG. All assays were developed using the TetraMethylBenzidine (TMB) Substrate Reagent Set (BD Optical Enzyme ImunoAssay [BDOptEIA]). ODs were measured at 450 nm.

### Synovial tissue histological analysis.

Formalin-fixed, paraffin-embedded (FFPE) 3 μm thick synovial tissue sections were used for both IHC and multiplex immunofluorescence (MIF). Sections were digitally scanned using the Nanozoomers S210 and S60 (Hamamatsu Photonics) and visualized with NDP.view 2 Software (Hamamatsu Photonics).

Lymphoid aggregates were identified in IHC using specific staining for CD20 (Dako, M0755), CD3 (Dako, M7254), and CD138 (Dako, M7228) (Agilent Technologies). Sections were counterstained with hematoxylin and mounted with Distyrene Plasticizer Xylene (DPX) mounting medium (MilliporeSigma).

MIF staining was performed using a tyramide signal amplification protocol (Invitrogen, Thermo Fisher Scientific). The following 3 primary antibodies were used: anti-HSP60 (Abcam, ab5478), anti-CD55 (Abcam, ab133684), and anti-CD90 (Abcam, ab133350). Slides were counterstained with DAPI and mounted with ProLong Antifade Mountant (Invitrogen, Thermo Fisher Scientific).

Quantitative digital image analyses were performed with QuPath software (42).

### CIA mouse model.

DBA/1 male mice (8–12 weeks of age) were purchased from Harlan Envigo and housed in the Biological Service Unit at the William Harvey Research Institute (QMUL). Detailed methods are reported in the Supplemental Methods.

### Synovial bulk RNA-Seq analysis.

Synovial bulk RNA-Seq analysis from the PEAC study (https://peac-mrc.mds.qmul.ac.uk/) and R4RA clinical trial was performed as previously published (26, 27).

### GeoMx DSP.

Synovial tissue from patients with RA from the R4RA cohort was profiled using the GeoMx DSP platform as published previously (27).

### Statistics.

Immunofluorescence colocalization analysis was performed using Pearson’s correlation coefficient with ImageJ (JACoP plugin, NIH) (23). For the arthritis score, a linear regression model using time and treatment and their interaction as fixed factors was used with the following formula: arthritis score ~ treatment + time + treatment × time. The F test *P* value from the treatment × time interaction term was reported. Statistical analysis was performed using GraphPad Prism, version 7 (GraphPad Software). One-way ANOVA was used for multiple comparisons. Correlations between SF and serum for anti-HSP60 Igs was calculated using Spearman’s rank correlation coefficient. Statistical significance for the comparison between RA and OA SF and ACPA^+^ and ACPA^–^ RA samples was assessed using unpaired, nonparametric tests. Anti-HSP60^+^ samples were defined for values higher than the mean ± 3 SD of the OA group for each Ig. A *P* value of less than 0.05 was considered statistically significant. RNA-Seq and longitudinal studies data were obtained using public data sets (http://peac.hpc.qmul.qc.uk/; ref. 26; and http://r4ra.hpc.qmul.ac.uk; refs. 27, 28). RNA-Seq methodology and statistical analysis using negative binomial general linear regression models and negative binomial general linear mixed models (GLMMs) for longitudinal sample analysis are described in full in these studies.

### Study approval.

Tissue, serum, and SF from patients with RA were obtained with informed consent (National Research Ethics Service Committee London, LREC 05/Q0703/198; REC 07/Q0605/29; REC ID: 22/WS/0147 – R4RA study). All experimental procedures involving animals were approved by the UK Home Office (project licenses PIL 70/23296 and P29EDC088).

### Data availability.

All supporting data from the study can be found in the Supporting Data Values file.

## Author contributions

EC, CP, and MB conceptualized the study. EC, MC, LJ, LFJ, MH, BJ, AAP, and MJL designed the study methodology. EC, MC, LJ, LFJ, KG, CC, GMG, FR, and MJL conducted formal analysis. IEC, MC, LJ, LFJ, MH, BJ, and AAP performed experiments. EC, MC, LJ, KG, MJL, and MB performed visualization studies. EC and MB acquired funding. EC and MB were responsible for project administration. EC and MB supervised the work. EC, MC, LJ, and MB wrote the original draft of the manuscript. EC, MC, LJ, LFJ, EP, CA, BJ, AAP, MJL, CP, and MB reviewed and edited the manuscript.

## Supplementary Material

Supplemental data

Unedited blot and gel images

Supporting data values

## Figures and Tables

**Table 1:**
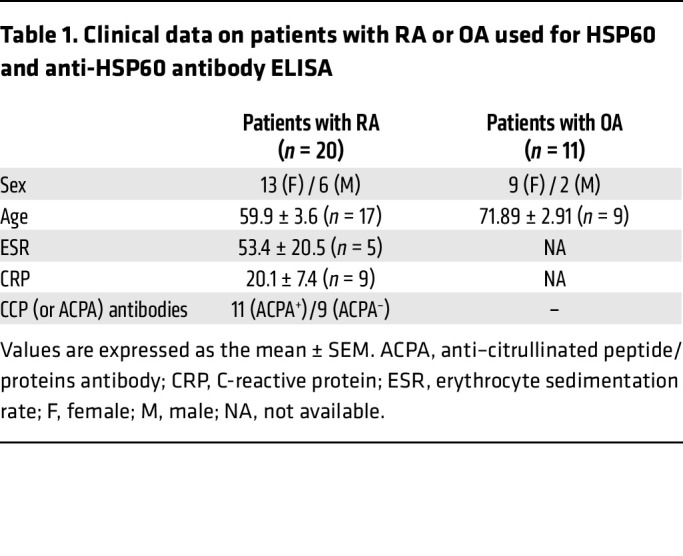
Clinical data on patients with RA or OA used for HSP60 and anti-HSP60 antibody ELISA

## Version 1 06/17/2024

Electronic publication

## References

[1] Bombardieri M (2017). Ectopic lymphoid neogenesis in rheumatic autoimmune diseases. Nat Rev Rheumatol.

[2] Corsiero E (2016). Single cell cloning and recombinant monoclonal antibodies generation from RA synovial B cells reveal frequent targeting of citrullinated histones of NETs. Ann Rheum Dis.

[3] Humby F (2009). Ectopic lymphoid structures support ongoing production of class-switched autoantibodies in rheumatoid synovium. PLoS Med.

[4] Bartok B, Firestein GS (2010). Fibroblast-like synoviocytes: key effector cells in rheumatoid arthritis. Immunol Rev.

[5] Bombardieri M (2011). A BAFF/APRIL-dependent TLR3-stimulated pathway enhances the capacity of rheumatoid synovial fibroblasts to induce AID expression and Ig class-switching in B cells. Ann Rheum Dis.

[6] Huber LC (2006). Synovial fibroblasts: key players in rheumatoid arthritis. Rheumatology (Oxford).

[7] McInnes IB, Schett G (2011). The pathogenesis of rheumatoid arthritis. N Engl J Med.

[8] Brennan FM, McInnes IB (2008). Evidence that cytokines play a role in rheumatoid arthritis. J Clin Invest.

[9] Hu F (2014). Toll-like receptors expressed by synovial fibroblasts perpetuate Th1 and th17 cell responses in rheumatoid arthritis. PLoS One.

[10] Ospelt C (2009). Expression, regulation, and signaling of the pattern-recognition receptor nucleotide-binding oligomerization domain 2 in rheumatoid arthritis synovial fibroblasts. Arthritis Rheum.

[11] Roelofs MF (2006). Identification of small heat shock protein B8 (HSP22) as a novel TLR4 ligand and potential involvement in the pathogenesis of rheumatoid arthritis. J Immunol.

[12] Fouani M (2022). Heat shock proteins alterations in rheumatoid arthritis. Int J Mol Sci.

[13] Holoshitz J (2010). A role for calreticulin in the pathogenesis of rheumatoid arthritis. Ann N Y Acad Sci.

[14] Tarr JM (2010). Extracellular calreticulin is present in the joints of patients with rheumatoid arthritis and inhibits FasL (CD95L)-mediated apoptosis of T cells. Arthritis Rheum.

[15] Spierings J, van Eden W (2017). Heat shock proteins and their immunomodulatory role in inflammatory arthritis. Rheumatology (Oxford).

[16] Mantej J (2019). Autoantibodies to heat shock proteins 60, 70, and 90 in patients with rheumatoid arthritis. Cell Stress Chaperones.

[17] Boog CJ (1992). Two monoclonal antibodies generated against human hsp60 show reactivity with synovial membranes of patients with juvenile chronic arthritis. J Exp Med.

[18] Corsiero E (2018). Characterization of a synovial B cell-derived recombinant monoclonal antibody targeting stromal calreticulin in the rheumatoid joints. J Immunol.

[19] Lambrecht S (2014). Heat-shock proteins in stromal joint tissues: innocent bystanders or disease-initiating proteins?. Rheumatology (Oxford).

[20] Tiller T (2008). Efficient generation of monoclonal antibodies from single human B cells by single cell RT-PCR and expression vector cloning. J Immunol Methods.

[21] Corsiero E (2014). Accumulation of self-reactive naïve and memory B cell reveals sequential defects in B cell tolerance checkpoints in Sjögren’s syndrome. PLoS One.

[22] Dunn KW (2011). A practical guide to evaluating colocalization in biological microscopy. Am J Physiol Cell Physiol.

[23] Bolte S, Cordelieres FP (2006). A guided tour into subcellular colocalization analysis in light microscopy. J Microsc.

[24] Bie AS (2020). An inventory of interactors of the human HSP60/HSP10 chaperonin in the mitochondrial matrix space. Cell Stress Chaperones.

[25] Renato GS (2013). Anti-citrullinated protein antibodies as novel therapeutic drugs in rheumatoid arthritis. J Clin Cell Immunol.

[26] Lewis MJ (2019). Molecular portraits of early rheumatoid arthritis identify clinical and treatment response phenotypes. Cell Rep.

[27] Rivellese F (2022). Rituximab versus tocilizumab in rheumatoid arthritis: synovial biopsy-based biomarker analysis of the phase 4 R4RA randomized trial. Nat Med.

[28] Humby F (2021). Rituximab versus tocilizumab in anti-TNF inadequate responder patients with rheumatoid arthritis (R4RA): 16-week outcomes of a stratified, biopsy-driven, multicentre, open-label, phase 4 randomised controlled trial. Lancet.

[29] Zhang F (2019). Defining inflammatory cell states in rheumatoid arthritis joint synovial tissues by integrating single-cell transcriptomics and mass cytometry. Nat Immunol.

[30] Srivastava P (2002). Roles of heat-shock proteins in innate and adaptive immunity. Nat Rev Immunol.

[31] Celis L (1997). Clonal expansion of mycobacterial heat-shock protein-reactive T lymphocytes in the synovial fluid and blood of rheumatoid arthritis patients. Arthritis Rheum.

[32] De Graeff-Meeder ER (1991). Recognition of human 60 kD heat shock protein by mononuclear cells from patients with juvenile chronic arthritis. Lancet.

[33] Huang MN (2010). The involvement of heat-shock proteins in the pathogenesis of autoimmune arthritis: a critical appraisal. Semin Arthritis Rheum.

[34] Tishler M, Shoenfeld Y (1996). Anti-heat-shock protein antibodies in rheumatic and autoimmune diseases. Semin Arthritis Rheum.

[35] Jang EJ (2013). Characterization of human anti-heat shock protein 60 monoclonal autoantibody Fab fragments in atherosclerosis: genetic and functional analysis. Mol Immunol.

[36] Yokota S (2006). Anti-HSP auto-antibodies enhance HSP-induced pro-inflammatory cytokine production in human monocytic cells via Toll-like receptors. Int Immunol.

[37] Kissel T (2020). Antibodies and B cells recognising citrullinated proteins display a broad cross-reactivity towards other post-translational modifications. Ann Rheum Dis.

[38] Reijm S (2021). Cross-reactivity of IgM anti-modified protein antibodies in rheumatoid arthritis despite limited mutational load. Arthritis Res Ther.

[39] Humby F (2019). Synovial cellular and molecular signatures stratify clinical response to csDMARD therapy and predict radiographic progression in early rheumatoid arthritis patients. Ann Rheum Dis.

[40] Holmdahl R (1986). Female sex hormones suppress development of collagen-induced arthritis in mice. Arthritis Rheum.

[41] Aletaha D (2010). 2010 Rheumatoid arthritis classification criteria: an American College of Rheumatology/European League Against Rheumatism collaborative initiative. Arthritis Rheum.

[42] Bankhead P (2017). QuPath: Open source software for digital pathology image analysis. Sci Rep.

