# Supplementary material for: Autoimmunity to stromal-derived autoantigens in rheumatoid ectopic germinal centers exacerbates arthritis and affects clinical response
Source: J Clin Invest. 2024 Apr 30;134(12):e169754. doi: 10.1172/JCI169754 (PMC11178537; doi:10.1172/JCI169754)
Supplement: Supplemental data [file jci-134-169754-s221.pdf]

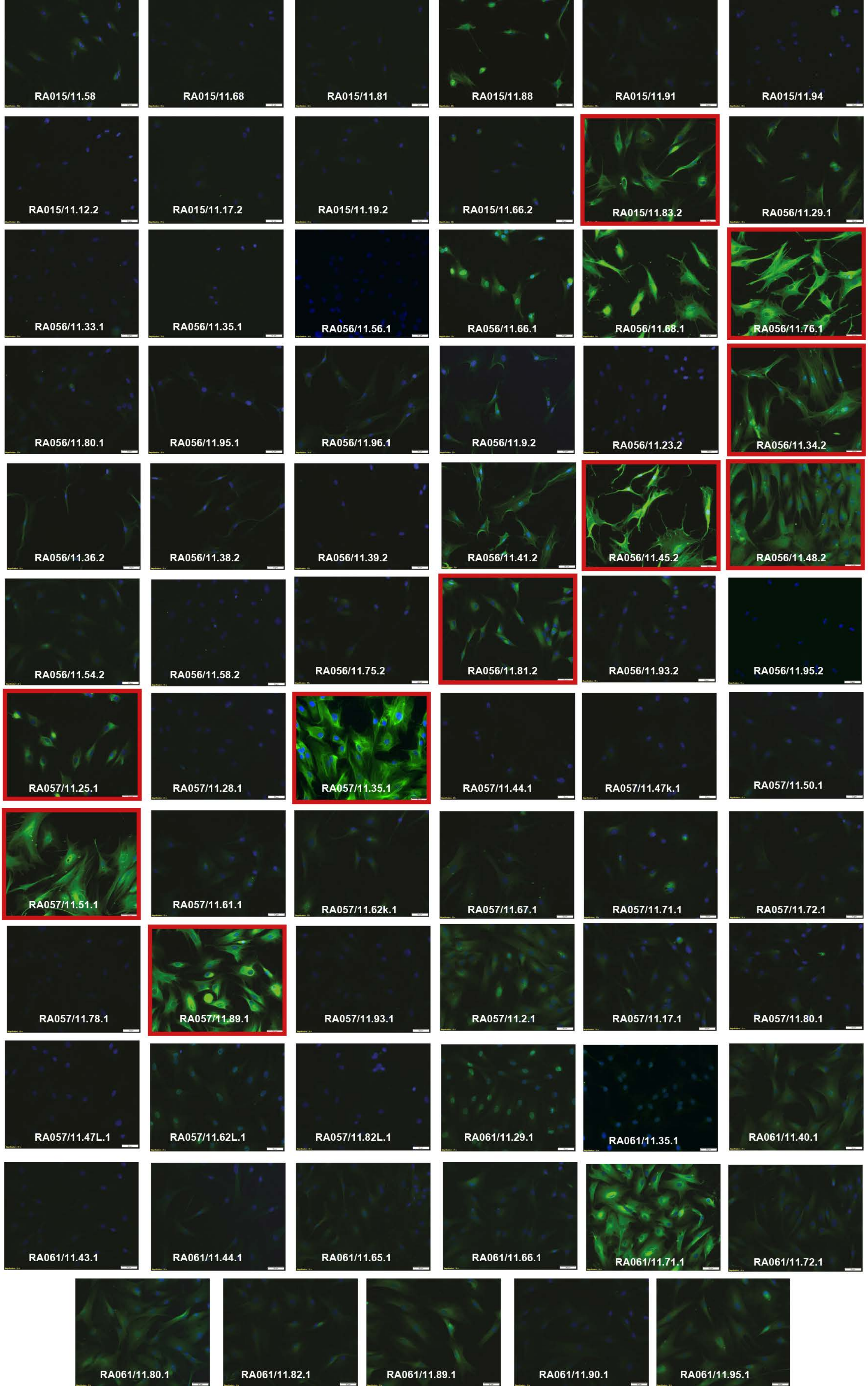
RA015/11.58
RA015/11.68
RA015/11.81
RA015/11.88
RA015/11.91
RA015/11.94
RA015/11.12.2
RA015/11.17.2
RA015/11.19.2
RA015/11.66.2
RA015/11.83.2
RA056/11.29.1
RA056/11.33.1
RA056/11.35.1
RA056/11.56.1
RA056/11.66.1
RA056/11.68.1
RA056/11.76.1
RA056/11.80.1
RA056/11.95.1
RA056/11.96.1
RA056/11.9.2
RA056/11.23.2
RA056/11.34.2
RA056/11.36.2
RA056/11.38.2
RA056/11.39.2
RA056/11.41.2
RA056/11.45.2
RA056/11.48.2
RA056/11.54.2
RA056/11.58.2
RA056/11.75.2
RA056/11.81.2
RA056/11.93.2
RA056/11.95.2
RA057/11.25.1
RA057/11.28.1
RA057/11.35.1
RA057/11.44.1
RA057/11.47k.1
RA057/11.50.1
RA057/11.51.1
RA057/11.61.1
RA057/11.62k.1
RA057/11.67.1
RA057/11.71.1
RA057/11.72.1
RA057/11.78.1
RA057/11.89.1
RA057/11.93.1
RA057/11.2.1
RA057/11.17.1
RA057/11.80.1
RA057/11.47L.1
RA057/11.62L.1
RA057/11.82L.1
RA061/11.29.1
RA061/11.35.1
RA061/11.40.1
RA061/11.43.1
RA061/11.44.1
RA061/11.65.1
RA061/11.66.1
RA061/11.71.1
RA061/11.72.1
RA061/11.80.1
RA061/11.82.1
RA061/11.89.1
RA061/11.90.1
RA061/11.95.1

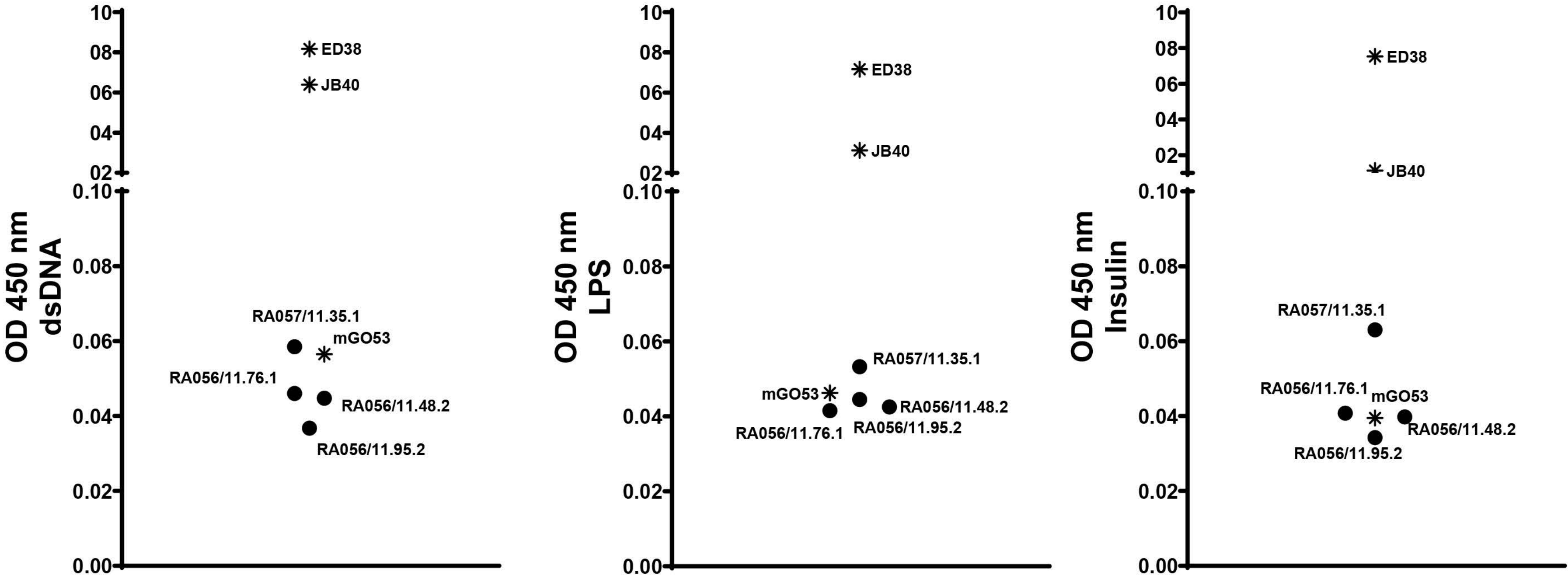

OD 450 nm dsDNA
10
08
06
04
02
0.10
0.08
0.06
0.04
0.02
0.00
ED38
JB40
RA057/11.35.1
mGO53
RA056/11.76.1
RA056/11.48.2
RA056/11.95.2
OD 450 nm LPS
ED38
JB40
RA057/11.35.1
mGO53
RA056/11.48.2
RA056/11.76.1
RA056/11.95.2
OD 450 nm Insulin
ED38
JB40
RA057/11.35.1
RA056/11.76.1
mGO53
RA056/11.48.2
RA056/11.95.2

A)

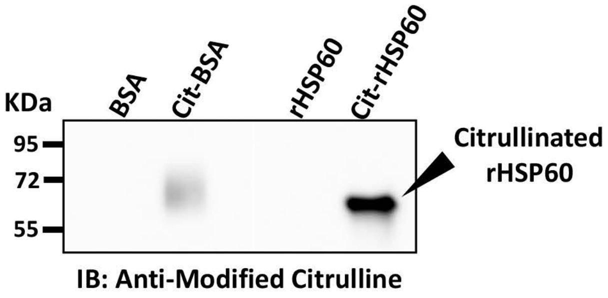
KDa
BSA
Cit-BSA
rHSP60
Cit-rHSP60
95
72
55
Citrullinated rHSP60
IB: Anti-Modified Citrulline

B)

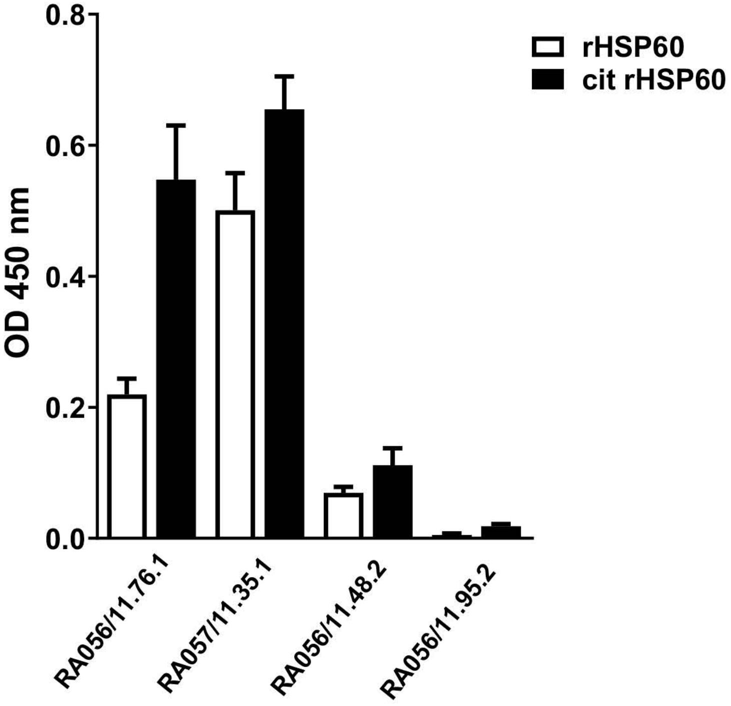
OD 450 nm
0.8
0.6
0.4
0.2
0.0
rHSP60
cit rHSP60
RA056/11.76.1
RA057/11.35.1
RA056/11.48.2
RA056/11.95.2

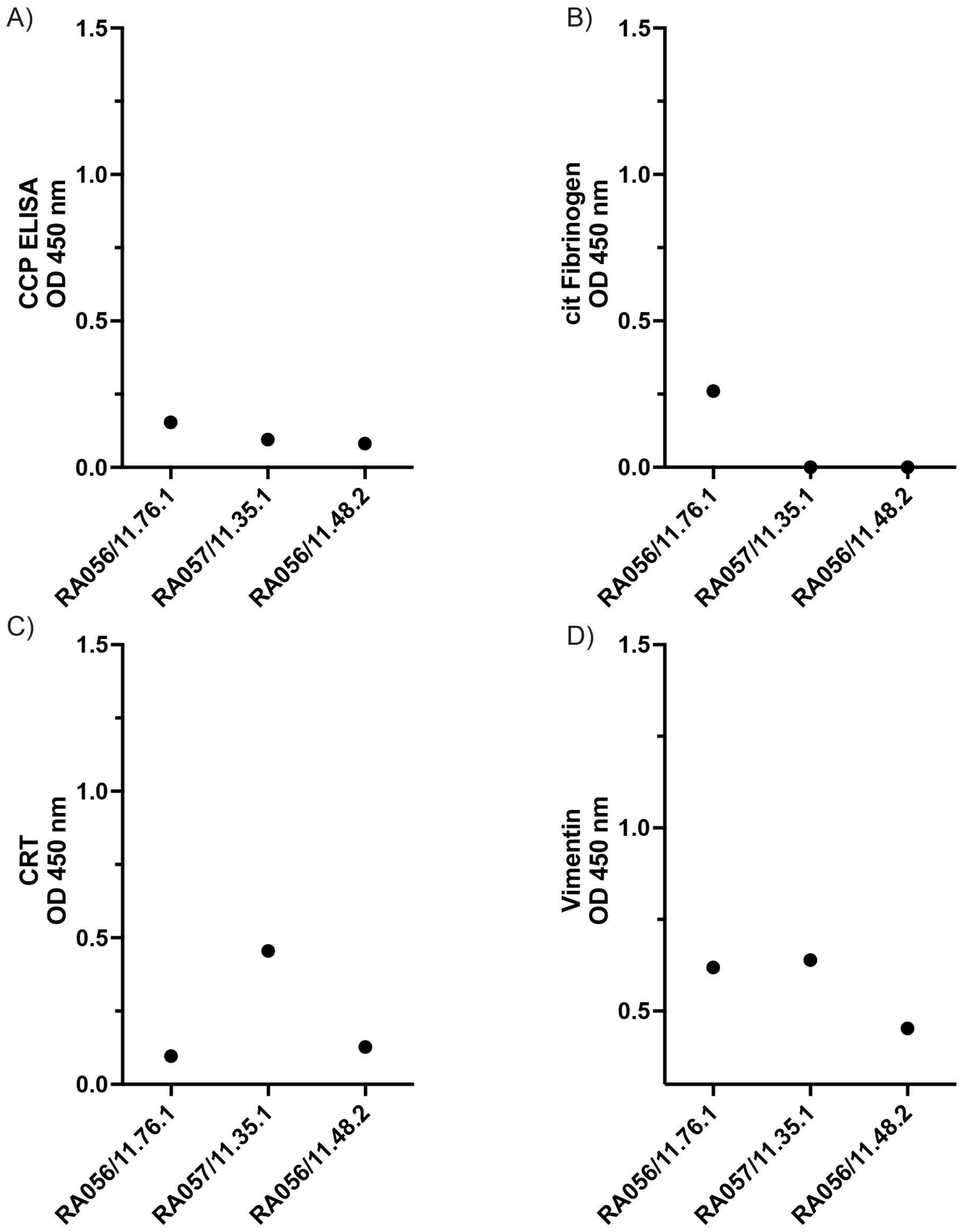

A)
1.5
1.0
0.5
0.0
CCP ELISA
OD 450 nm
RA056/11.76.1
RA057/11.35.1
RA056/11.48.2
B)
1.5
1.0
0.5
0.0
cit Fibrinogen
OD 450 nm
RA056/11.76.1
RA057/11.35.1
RA056/11.48.2
C)
1.5
1.0
0.5
0.0
CRT
OD 450 nm
RA056/11.76.1
RA057/11.35.1
RA056/11.48.2
D)
1.5
1.0
0.5
Vimentin
OD 450 nm
RA056/11.76.1
RA057/11.35.1
RA056/11.48.2

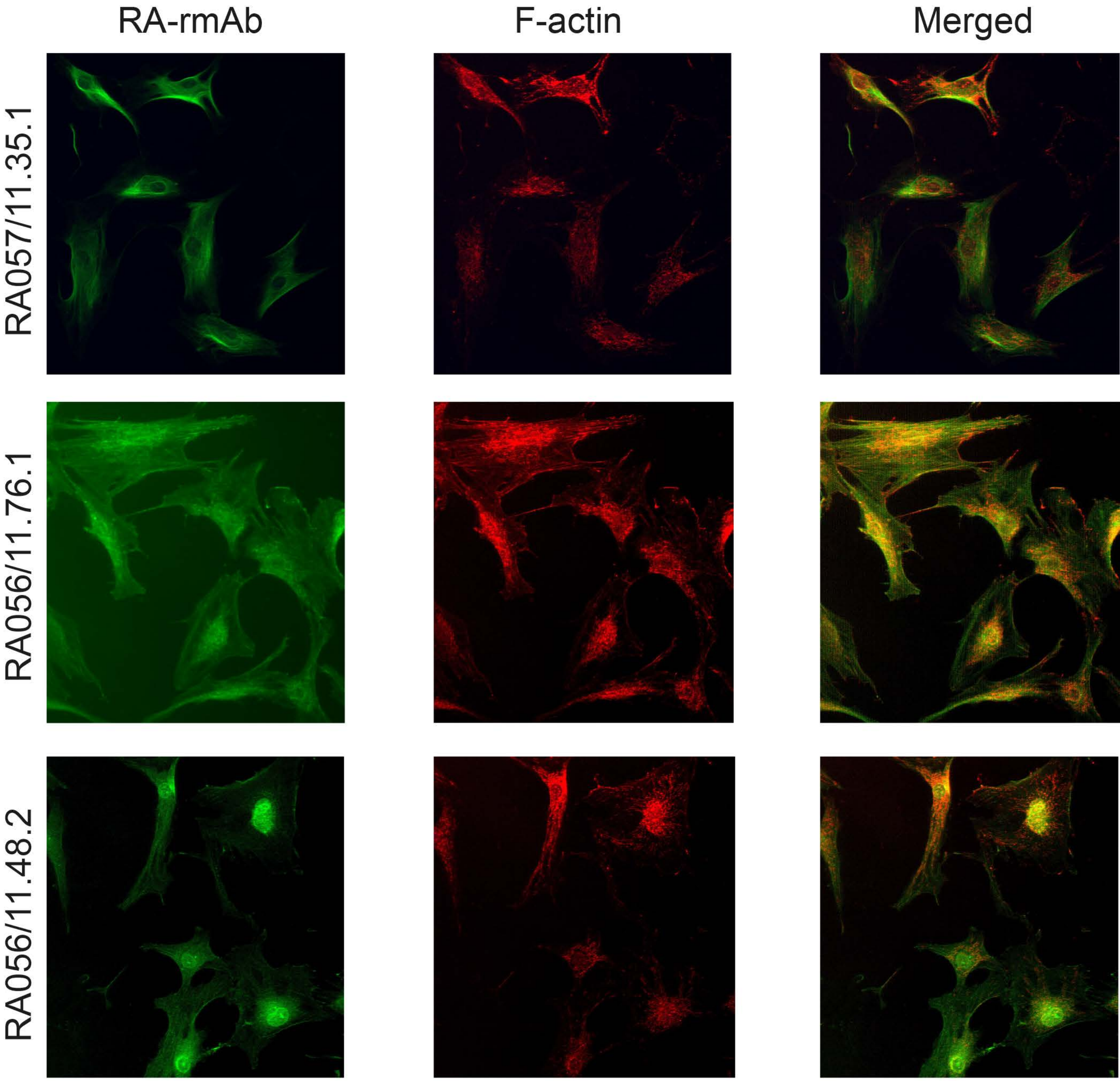
RA-rmAb
F-actin
Merged
RA057/11.35.1
RA056/11.76.1
RA056/11.48.2

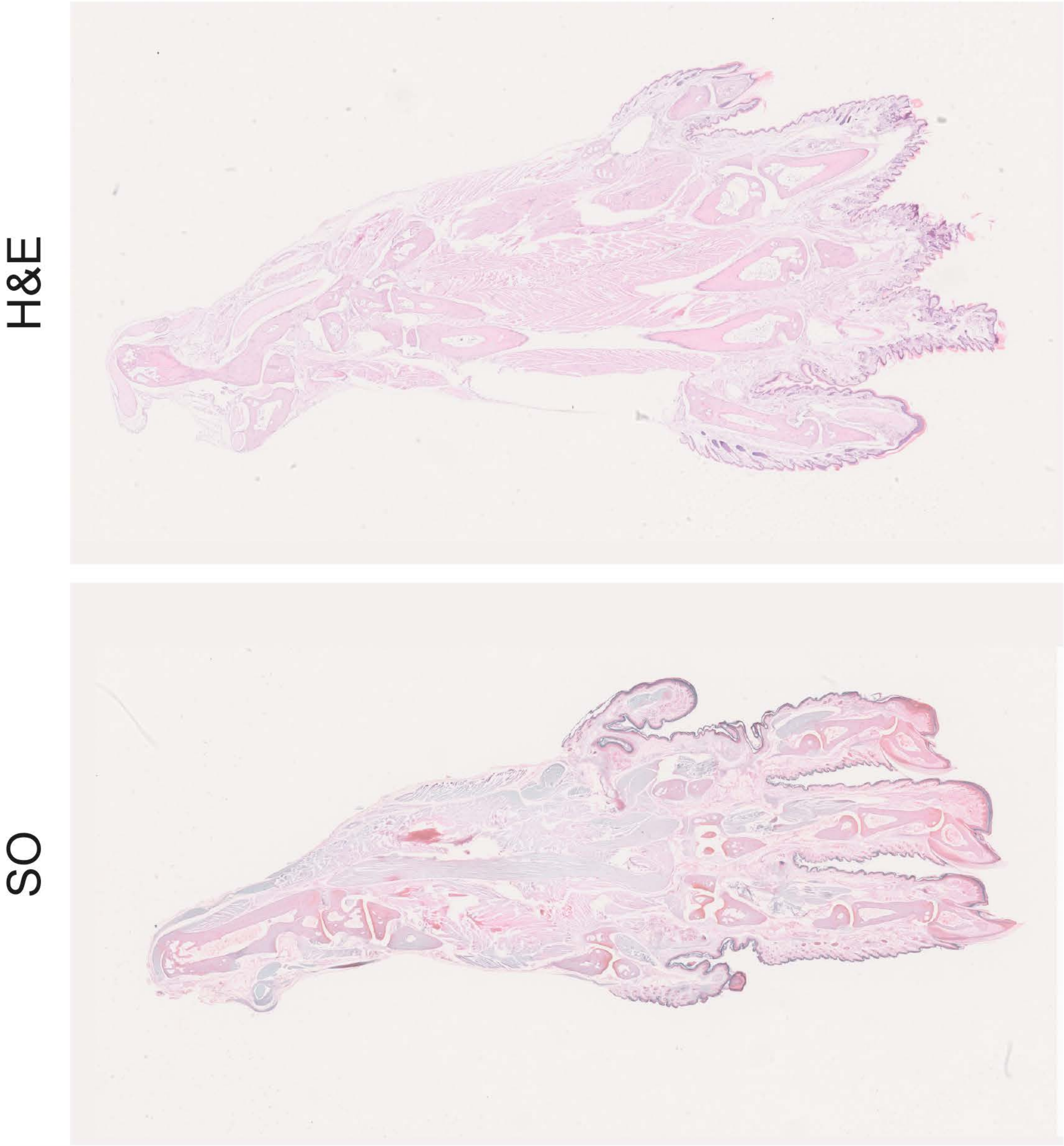
H&E
SO

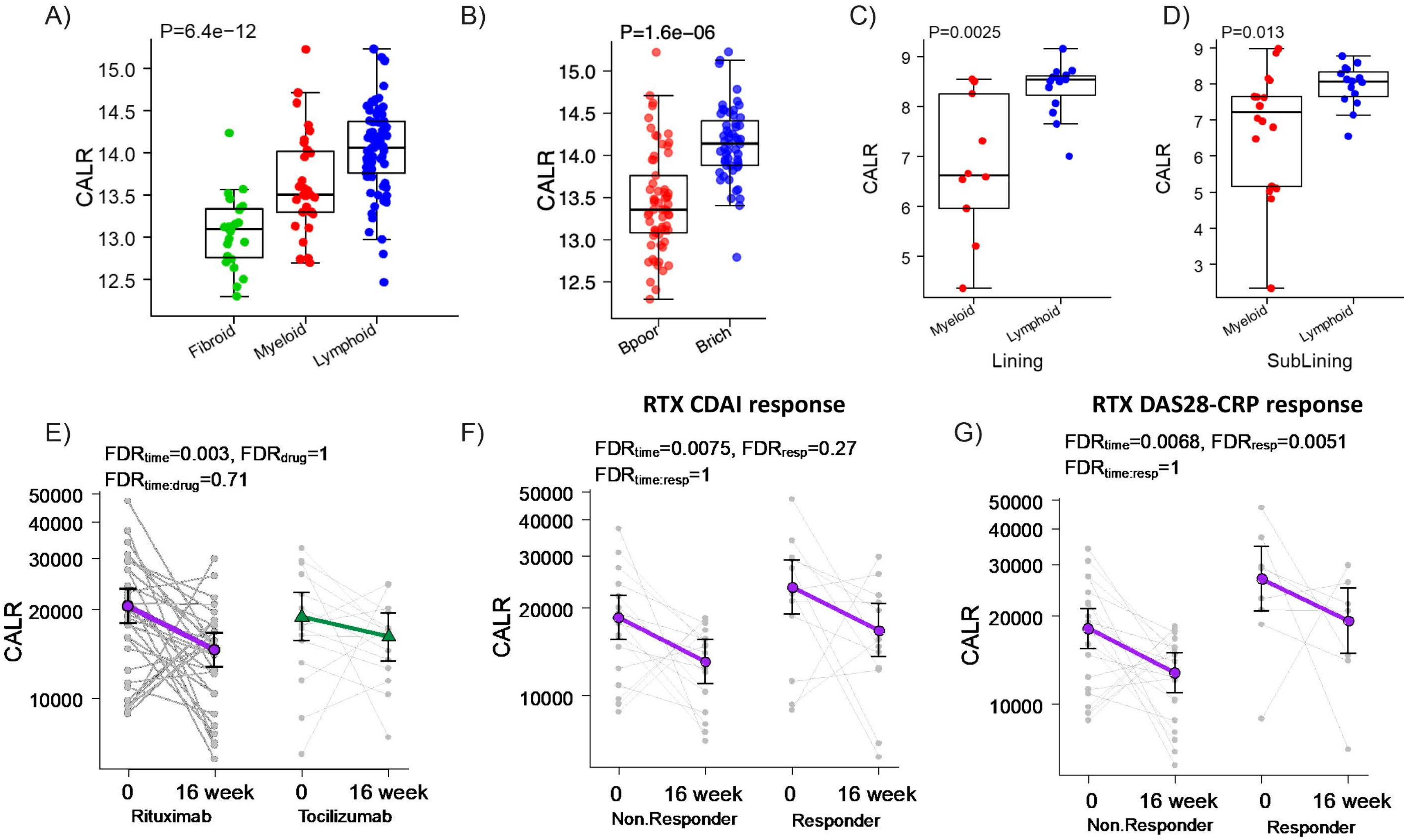

A)
P=6.4e−12
CALR
15.0
14.5
14.0
13.5
13.0
12.5
Fibroid
Myeloid
Lymphoid
B)
P=1.6e−06
CALR
Bpoor
Brich
C)
P=0.0025
CALR
Myeloid
Lymphoid
Lining
D)
P=0.013
CALR
Myeloid
Lymphoid
SubLining
RTX CDAI response
RTX DAS28-CRP response
E)
FDRtime=0.003, FDRdrug=1
FDRtime:drug=0.71
50000
40000
30000
20000
10000
0
16 week
Rituximab
Tocilizumab
F)
FDRtime=0.0075, FDRresp=0.27
FDRtime:resp=1
Non.Responder
Responder
G)
FDRtime=0.0068, FDRresp=0.0051
FDRtime:resp=1

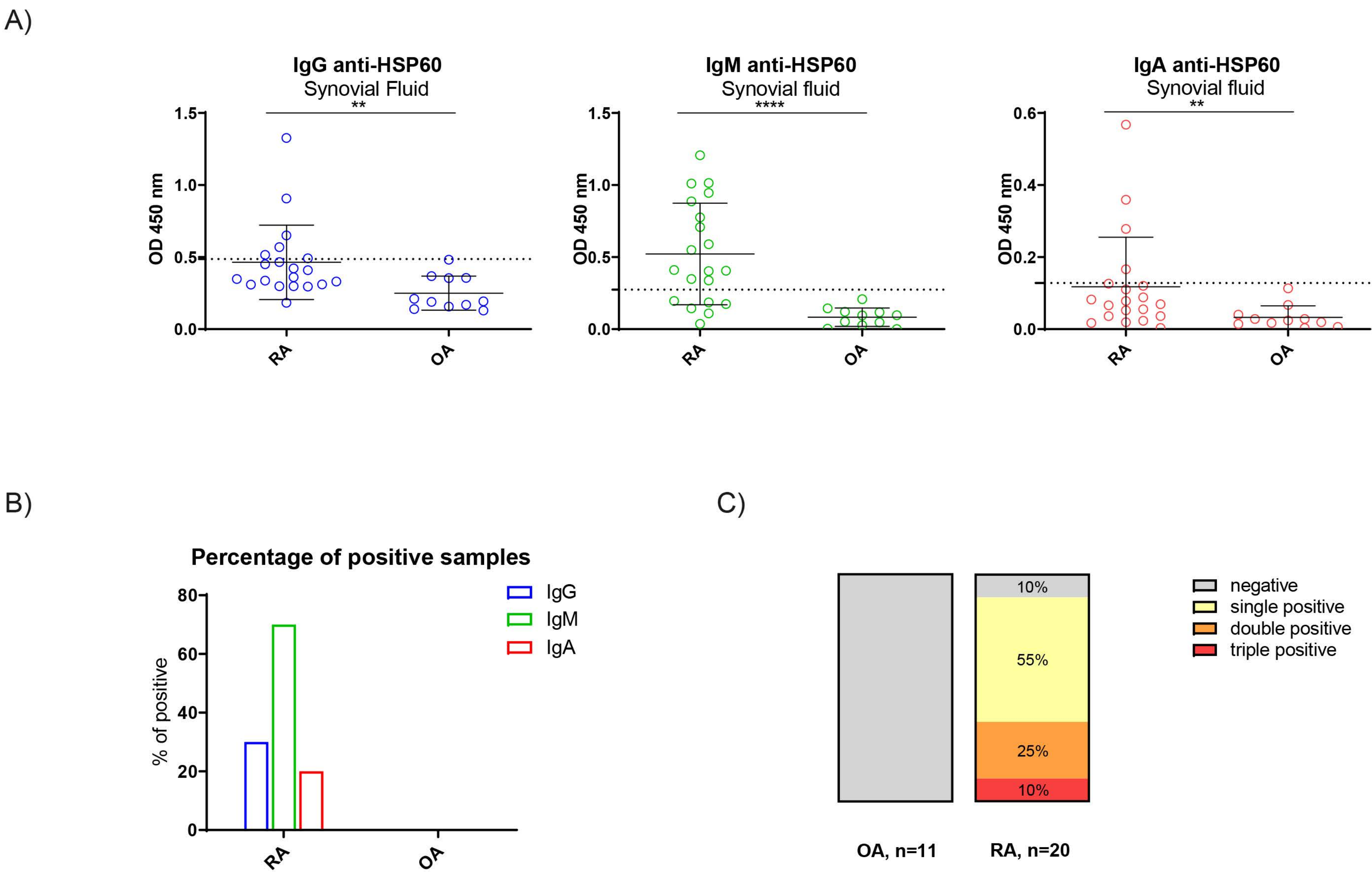
A)
IgG anti-HSP60
Synovial Fluid
**
OD 450 nm
1.5
1.0
0.5
0.0
RA
OA
IgM anti-HSP60
Synovial fluid
****
OD 450 nm
1.5
1.0
0.5
0.0
RA
OA
IgA anti-HSP60
Synovial fluid
**
OD 450 nm
0.6
0.4
0.2
0.0
RA
OA
B)
Percentage of positive samples
% of positive
80
60
40
20
0
IgG
IgM
IgA
RA
OA
C)
10%
55%
25%
10%
negative
single positive
double positive
triple positive
OA, n=11
RA, n=20

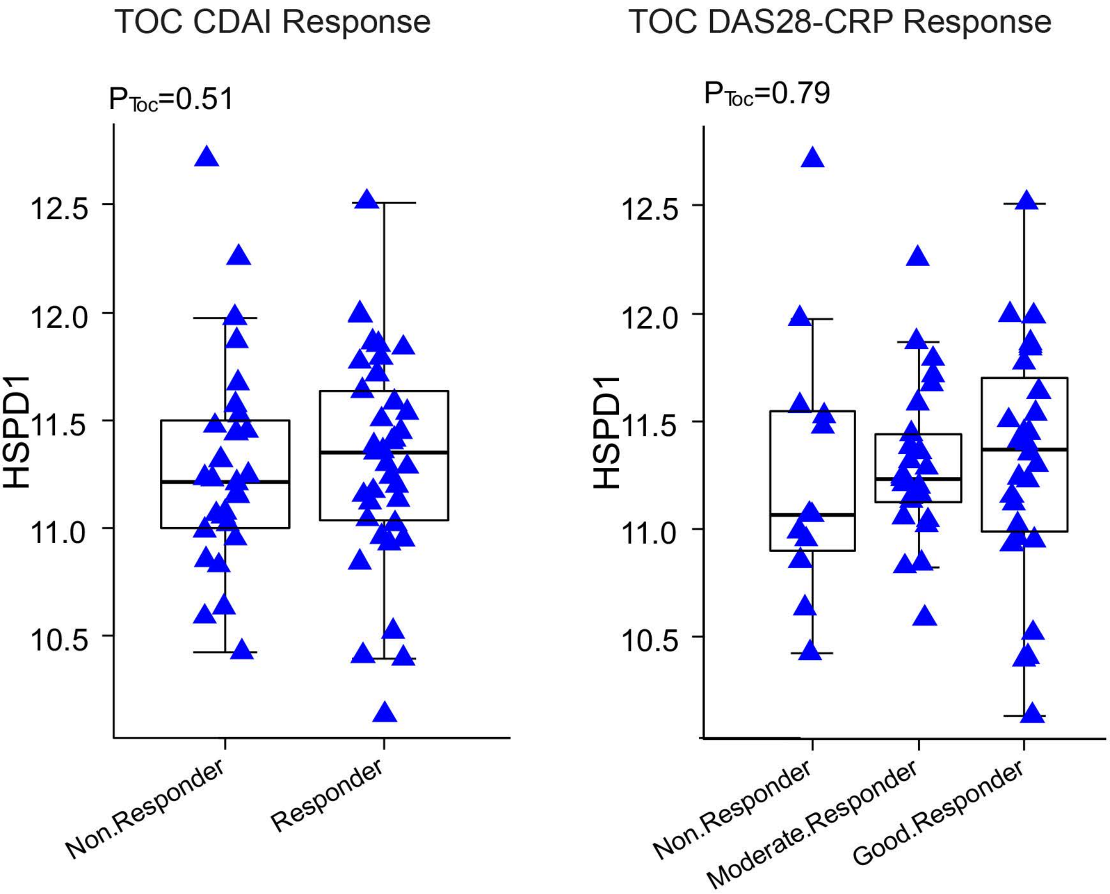

TOC CDAI Response
TOC DAS28-CRP Response
PToc=0.51
PToc=0.79
HSPD1
12.5
12.0
11.5
11.0
10.5
Non.Responder
Responder
Moderate.Responder
Good.Responder

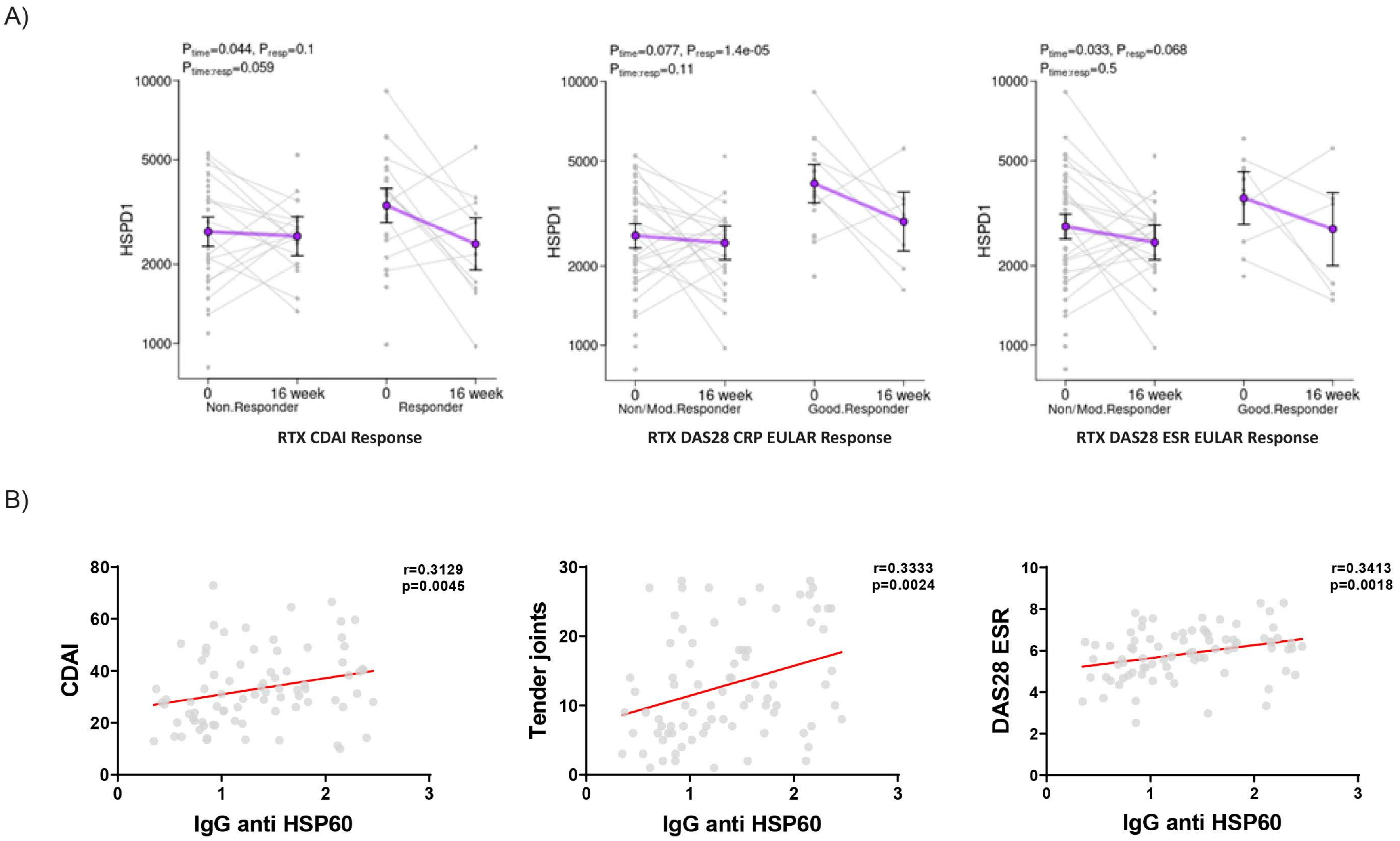
A)
P_time=0.044, P_resp=0.1
P_time:resp=0.059
10000
5000
2000
1000
HSPD1
0
16 week
Non.Responder
0
16 week
Responder
RTX CDAI Response
P_time=0.077, P_resp=1.4e-05
P_time:resp=0.11
Non/Mod.Responder
Good.Responder
RTX DAS28 CRP EULAR Response
P_time=0.033, P_resp=0.068
P_time:resp=0.5
Non/Mod.Responder
Good.Responder
RTX DAS28 ESR EULAR Response
B)
r=0.3129
p=0.0045
CDAI
IgG anti HSP60
r=0.3333
p=0.0024
Tender joints
IgG anti HSP60
r=0.3413
p=0.0018
DAS28 ESR
IgG anti HSP60

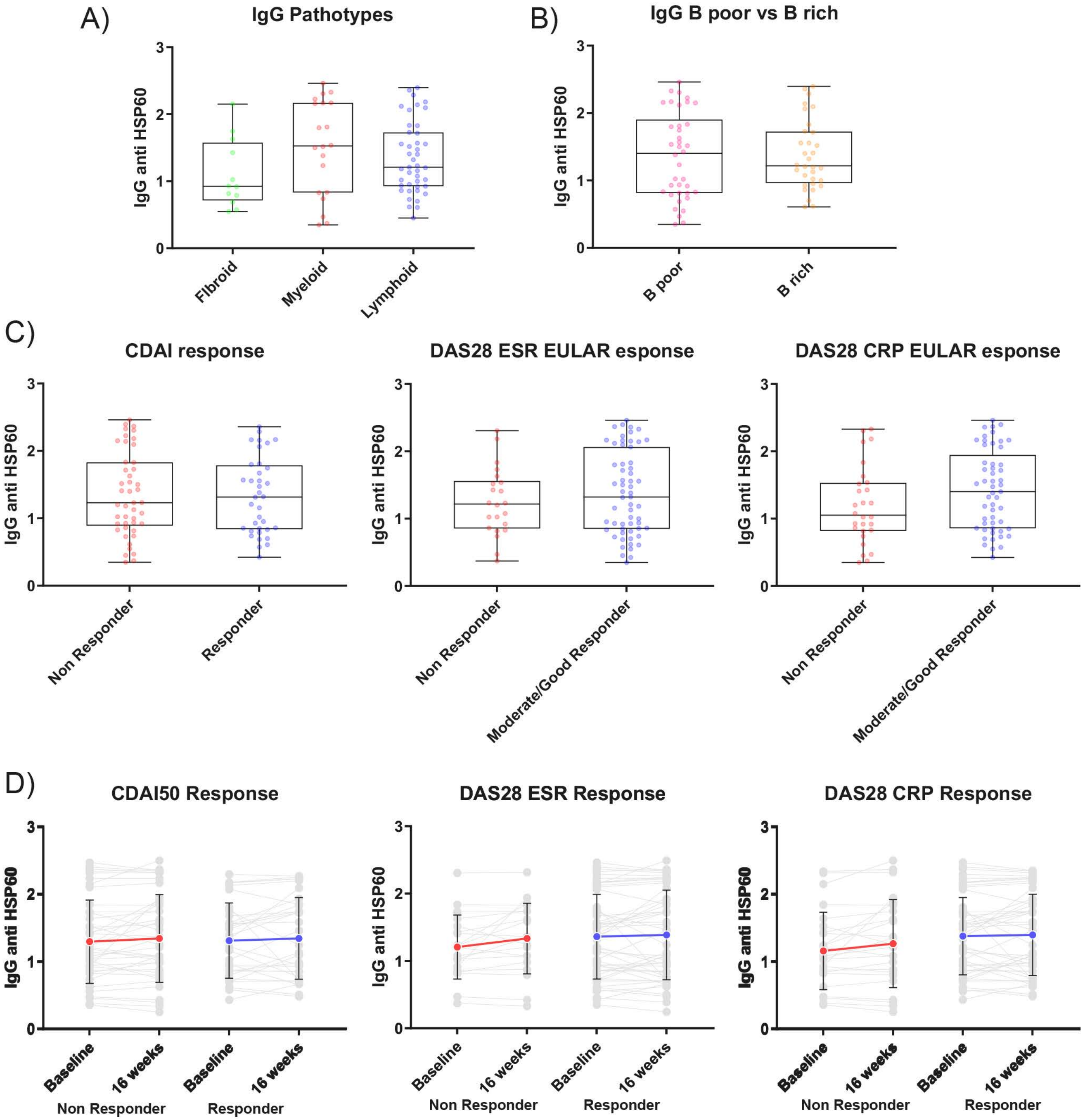

A)
IgG Pathotypes
IgG anti HSP60
Fibroid
Myeloid
Lymphoid
B)
IgG B poor vs B rich
B poor
B rich
C)
CDAI response
DAS28 ESR EULAR esponse
DAS28 CRP EULAR esponse
Non Responder
Responder
Moderate/Good Responder
D)
CDAI50 Response
DAS28 ESR Response
DAS28 CRP Response
Baseline
16 weeks
Non Responder
Responder

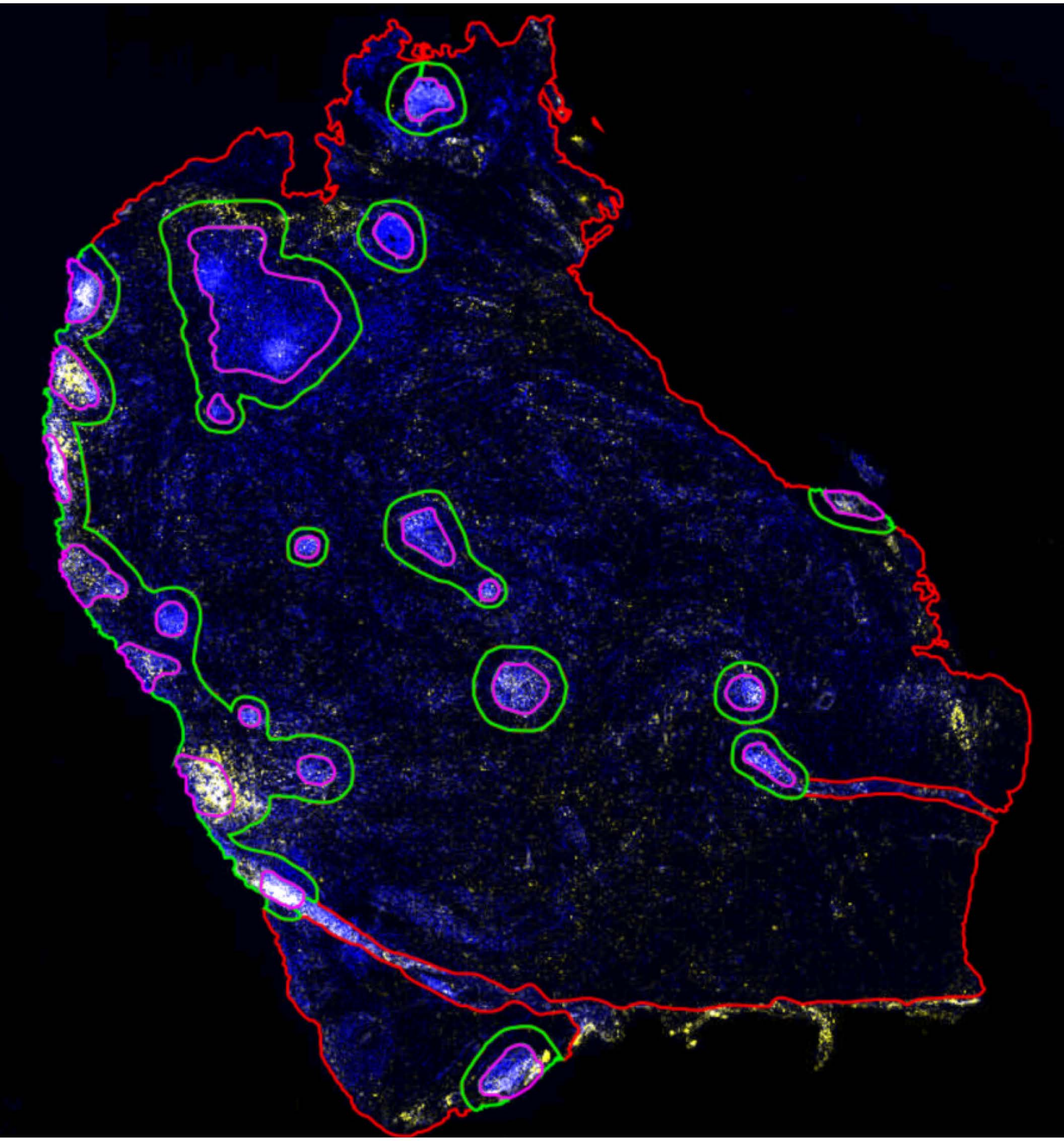

Supplementary Materials

Generation of RA-rmAbs from ELS+ RA synovial tissue

RA-rmAbs were generated from single synovial CD19+ B cells, as previously reported (2, 18). Briefly, after single cell FACS sorting, cDNA from single B cells was amplified by nested PCR using IgV gene-specific primers. Amplified Ig VH and VL genes were then cloned and expressed as full IgG1 recombinant monoclonal antibodies displaying identical specificity of the original B cells. All plasmids encoding both the VH and VL genes were prepared using endotoxin free MIDI kit (Macherey-Nagel).

Isolation of fibroblast-like synoviocytes from RA joints

Fibroblast-like synoviocytes (FLS) were obtained from synovial tissue, as previously described (5). At 90% confluent FLS were passaged 1:3 using 0.25% trypsin/EDTA (Sigma, UK). Culture medium (DMEM/F-12 GlutaMAX + 10% FBS + Antibiotic Antimycotic Solution) was replaced every 3 to 4 days. FLS were used after passage 4 to avoid any contamination from synovial macrophages and up to passage 8.

Immunoprecipitation experiments

Immunoprecipitation (IP) was performed by mixing equal amounts of RA-mAbs and recombinant human rhHSP60 (Abcam) in NP40 buffer (25 mM Tris-HCl pH 7.5, 137 mM NaCl, 10% Glycerol, 1% NP40, 1 mM EDTA) on a rotary shaker, overnight at 4°C. Protein A Sepharose beads (Cytiva) were added to the mixture and incubated on a rotary shaker for 1h at 4°C. After centrifugation and washing with cold NP40 buffer, the immunoprecipitates were eluted with 2X Laemmli buffer and resolved using SDS-PAGE and transferred to a nitrocellulose membrane (GE Healthcare Life Science). The blocking was performed in 5% (w/v) non-fat dry milk in TBST (TBS plus 0.1% Tween-20) for 1h, followed by incubation with a mouse anti-

human HSP60 (Abcam, ab5478) in blocking solution for 2h. After washing, the membrane was incubated with goat anti-mouse IgG peroxidase (Abcam, ab97040) for 1h. The membrane was washed again and incubated for 1 min in Clarity Western ECL substrate (BioRad). The blots were then developed using Hyperfilm ECL (GE Healthcare Life Science).

For the immunoprecipitation, a preclearing step was performed by incubating RA-FLS protein extract and Protein A Sepharose beads in NP40 buffer for 1h at 4°C under rotation. After centrifugation, the supernatant was recovered and transferred to a new tube and RA-rmAb was added and incubated overnight at 4°C in constant rotation. Protein A Sepharose beads were added to the sample and incubated for 1h. After washing, captured immunocomplexes were eluted with 2X Laemmli buffer and resolved using SDS-PAGE.

Liquid chromatography–tandem mass spectrometry analysis

For mass spectrometry analysis, RA-FLS cells were seeded and cultured in 150 mm dishes until they reached 80-90% of confluence. Cells were washed once in cold 1X PBS and lysed in NP40 buffer supplemented with Protease Inhibitor Cocktail (Sigma) and phosphatase inhibitors (PhosStop, ROCHE). Protein concentration was measured using the BCA Protein Assay Kit (Themo Scientific). Following the electrophoresis, the gel was stained using SimplyBlue SafeStain (Invitrogen), and a band around 60 kDa was cut and analysed by mass spectrometry. In-gel reduction, alkylation, and digestion with trypsin were performed on the excised gel bands prior to subsequent analysis by mass spectrometry. Cysteine residues were reduced with DTT and derivatized by treatment with iodoacetamide to form stable carbamidomethyl derivatives. Trypsin digestion was carried out overnight at RT after initial incubation for 2h at 37°C. Peptides were extracted from the gel pieces by a series of acetonitrile and aqueous washes. The extract was pooled with the initial supernatant and lyophilized. The sample was then resuspended in 50 mM ammonium bicarbonate to be analysed by liquid

chromatography–tandem mass spectrometry (LC-MS/MS). Chromatographic separation was performed using an EASY-nLC system (Thermo Fisher Scientific). Peptides were resolved by reversed-phase chromatography on a 75-mm C18 column using a three-step linear gradient of acetonitrile in 0.1% formic acid. The gradient was delivered to elute the peptides at a flow rate of 300 ml/min over 60 min. The eluate was ionized by electrospray ionization using an Orbitrap Velos Pro (Thermo Fisher Scientific) operating under Xcalibur v2.2. The instrument was programmed to acquire in automated data-dependent switching mode, selecting precursor ions based on their intensity for sequencing by collision-induced fragmentation using a Top20 CID method. The tandem mass spectrometry (MS/MS) analyses were conducted using collision energy profiles that were chosen based on the mass-to-charge ratio and the charge state of the peptide. Raw mass spectrometry data were processed into peak list files using Proteome Discoverer (v1.4; Thermo Fisher Scientific). Processed raw data were searched using the Mascot search algorithm (www.matrixscience.com) against the Uniprot database using All Taxonomy and Human Taxonomy.

Immunofluorescence microscopy on FLS

FLS were seeded at $1x10^4$ cells/200 µl onto cover slides. After 24 h, cells were washed in 1X PBS and fixed using 4% (final concentration) paraformaldehyde (PFA) or acetone: methanol (1:1). After washing in Tris-Buffered Saline (TBS) and blocking with serum-free protein block (DAKO), RA-rmAbs were diluted at 50 µg/ml in antibody diluent (DAKO) and applied for 1h at RT. After washing with 1X TBS, Alexa-488 goat anti-human IgG (Invitrogen, A11013) was applied for 1h RT. For HSP60 co-staining, a mouse anti-human HSP60 was added for 1h at RT followed by Alexa-555 goat-anti mouse IgG (Invitrogen, A21147). For F-Actin co-staining, a mouse anti-human F-Actin (Abcam, ab205) was added for 1h at RT followed by Alexa-647 donkey anti-Human IgG (Invitrogen, A21445). 4’, 6- DiAmidino-2-PhenylIndole (DAPI)

(Invitrogen) was added to visualize the nuclei. All sections were imaged using LSM 710 confocal microscope.

Quantitative digital image analysis

Quantitative digital image analysis was performed with QuPath software (**Supplementary Figure S12**). Lymphoid aggregates (in magenta) were defined based on CD20 expression in IHC and size, shape and numbers of the nuclei in MIF. The 100-200 µm area surrounding the aggregates was classified as peri-aggregate area (in green). The extension of each peri-aggregate region was defined based on the size of each aggregate and/or the presence of two or more of them. The rest of the section was defined and non-aggregate area (in red). For each region of interest, the percentage of HSP60 area was measured. Three lymphoid treatment-naïve RA synovial tissues were compared after staining two or three non-sequential sections for each patient.

Collagen-induce arthritis mouse model

CIA was induced by intradermal immunization with 200 μg/mouse bovine collagen-type-II (CII) (MDBioscience) in Complete Freund Adjuvant on day 0 with a boost at day 21 with 200 μg/mouse CII in Incomplete Freund Adjuvant, as by routine protocol [40]. Selected anti-FLS rmAbs cocktail (n=3) (controls: PBS/hIgG-treated mice; anti-histone rmAbs n=3) was administered i.p. four times at interval of 7-10 days with the 1st injection at day 16 (100 μg/mouse - 10 animals/group). Mice were evaluated every other day for arthritis incidence/severity until culling (up to day 55). Every inflamed main digit scored one and each ankle scored one, thus, a maximal score for each animal was 22. After culling, paws were imaged by micro-computed-tomography (micro-CT) to analyse bone loss and they were paraffin-embedded for histological evaluation. Hind paws were used for histological analysis. Tissue was fixed for 2 days in 4% formaldehyde, decalcified using an EDTA + 10%

paraformaldehyde decalcifier solution for 14 days, and subsequently dehydrated and embedded in paraffin. Sections of 5 μm were mounted on SuperFrost slides. Haematoxylin and eosin (HE) staining was performed to study joint inflammation. To study proteoglycan (PG) depletion from the cartilage matrix, sections were stained with Safranin O (SO), followed by counterstaining with fast green.

Micro-CT Scanning

Samples were wrapped in plastic film prior to scanning to prevent drying and scanned using a Skyscan 1172F (Bruker, Belgium). Mouse femora were scanned with X-ray settings 50kV and 200μA, using an aluminium 0.5mm filter and exposure time 960ms using a pixel size of 5μm. Projection images were reconstructed into tomograms using NRecon 1.7.3.1 (Bruker, Belgium) and repositioned using Dataviewer 1.5.4 (Bruker, Belgium) before volume rendering and image acquisition in CTVox 3.3.0 (Bruker, Belgium).

Citrullination of HSP60 in vitro

hrHSP60 protein was incubated with rabbit skeletal muscle PAD (7.5 U/mg) in 0.1 M Tris-HCl (pH 7.4), 10 mM CaCl2, and 5 mM DTT for 2 h at 50°C. After incubation, HSP60 was stored at -20°C. Citrullination was confirmed by Western blot analysis using an Anti-Citrulline (Modified) Detection Kit (Merck Millipore) following the manufacturer's instruction (**Supplementary Figure S3a**).

Characterization of polyreactivity by ELISA

To test the reactivity against different allo- and auto-antigens, supernatants were tested for polyreactivity against double-stranded DNA (dsDNA), lipopolysaccharide (LPS) and insulin by ELISA, as previously reported (2, 20, 21). Antibodies that reacted against at least two structurally diverse self- and non-self-antigens were defined as polyreactive. Internal controls for polyreactivity were added on each plate consisting of the recombinant monoclonal

antibodies mGO53 (negative), JB40 (low polyreactive), and ED38 (highly polyreactive) as previously reported (20).

Supplementary Tables

RA-rmAb	VH	D	HJ	(-)	CDR3(aa)	(+)	Length
RA056/11.76.1	1-69	3-3	5	2	VRITIFGVVMVKSDNWFDP	2	19
RA056/11.48.2	3-15	2-21	5	2	HFESCGGDCSNW	1	12
RA057/11.35.1	4-34	6-13	4	1	GWAYSSSWYRRMISFDY	2	17
**RA-rmAb**	**κ/λ**	**Vκ**	**Jκ**	**(-)**	**CDR3**	**(+)**	**Length**
RA056/11.76.1	κ	3-15	2	2	QQYNNLYT	3	9
RA056/11.48.2	κ	1-39	2	0	QQSYSTPYT	0	9
RA057/11.35.1	κ	3-20	2	0	QQHGSSPYT	1	9

Supplementary Table S1. V(D)J gene usage of RA056/11.76.1, RA056/11.48.2 and RA057/11.35.1. (-) / (+), negative / positive charges; CDR = complementary determining region

RA-rmAb Heavy Chain	Isotype	V-REGION Nb of mutations	FR1-IMGT Nb of mutations	CDR1-IMGT Nb of mutations	FR2-IMGT Nb of mutations	CDR2-IMGT Nb of mutations	FR3-IMGT Nb of mutations	CDR3-IMGT Nb of mutations
RA056/11_76.1_HC	γ	1	1	0	0	0	0	0
RA056/11_48.2_HC	μ	0	0	0	0	0	0	0
RA057/11_35.1_HC	μ	4	4	0	0	0	0	0
**RA-rmAb Light Chain**		**V-REGION Nb of mutations**	**FR1-IMGT Nb of mutations**	**CDR1-IMGT Nb of mutations**	**FR2-IMGT Nb of mutations**	**CDR2-IMGT Nb of mutations**	**FR3-IMGT Nb of mutations**	**CDR3-IMGT Nb of mutations**
RA056/11_76.1_KC		6	6	0	0	0	0	0
RA056/11_48.2_KC		1	0	0	0	0	1	0
RA057/11_35.1_KC		11	1	1	1	0	6	2

Supplementary Table S2. V-region number of mutations. Number of mutations in the variable region of the heavy chain and light chain for the RA-rmAbs, RA056/11.76.1, RA056/11.48.2 and RA057/11.35.1. Isotype is shown for each antibody.

Supplementary Figure Legends

Supplementary Figure S1. RA-rmAbs reactivity towards RA-FLS.

Representative immunofluorescence pictures of RA-FLS incubated with the RA-rmAbs (n=71) showing immunoreactivity of 10 RA-rmAbs (highlighted in red) towards FLS-derived antigens only (green). Nuclei were stained with DAPI (blue).

Supplementary Figure S2. Polyreactivity analysis of the RA-rmAbs.

RA056/11.76.1, RA056/11.48.2 and RA057/11.35.1 were tested for reactivity with dsDNA (left), LPS (middle) and insulin (right) by ELISA. Each graph shows the reactivity at a concentration of 1 μg/ml and it shows the result of two independent experiments. Data points represent individual antibodies. Internal controls for polyreactivity are shown in each graph and include mGO53 (negative (20)), JB40 (low polyreactive (20)), and ED38 (highly polyreactive (20)).

Supplementary Figure S3. RA-rmAbs reactivity towards citrullinated HSP60

(a) Western blot using an anti-citrulline (modified) detection kit showing in vitro citrullination of rHSP60 by PAD enzyme. BSA and cit-BSA was used as control. **(b)** RA056/11.76.1, RA057/11.35.1 and RA056/11.48.2 immunoreactivity toward deiminated (white bar) and citrullinated HSP60 (black bar). Results are expressed as absorbance at 450 nm.

Supplementary Figure S4. RA-rmAbs reactivity towards cyclic citrullinated peptide (CCP), citrullinated fibrinogen, calreticulin (CRT) and vimentin.

RA056/11.76.1, RA056/11.48.2 and RA057/11.35.1 were tested in ELISA for their reactivity with **(a)** CCP, **(b)** cit-fibrinogen, **(c)** CRT and **(d)** vimentin. All RA-rmAbs were tested at a concentration of 10 μg/ml.

Supplementary Figure S5. RA-rmAbs and F-Actin immunofluorescence staining.

Representative immunofluorescence pictures showing staining for F-Actin (red) and RA-rmAbs (green). Magnification 20X.

Supplementary Figure S6. Anti-histones RA-rmAbs in CIA.

Representative IHC (top) and SO (bottom) images of anti-histones RA-rmAbs treated mice.

Supplementary Figure S7. Calreticulin expression and analysis of pre- and post-rituximab treatment synovial biopsies.

(a) Synovium *CALR* gene evaluation compared across histological pathotype in established RA patients. **(b)** Synovium *CALR* gene expression in patients stratified by histology into B-cell poor and B-cell rich. *CALR* differentially expressed in the lining **(c)** and sublining **(d)** area in the lympho-myeloid (lymphoid) vs diffuse-myeloid (myeloid) pathotypes using GeoMx DSP data. **(e)** *CALR* RNA-seq counts assessed at baseline and 16-weeks following rituximab and tocilizumab treatment in paired synovial biopsies. **(f) (g)** *CALR* counts assessed at baseline and 16-weeks following rituximab treatment in paired synovial biopsies. CDAI 50% improvement, DAS28-CRP EULAR response were used to assess the clinical response.

Supplementary Figure S8. Expression of anti-HSP60 antibodies in synovial fluid of RA and OA patients.

(a) IgG, IgM and IgA-binding to HSP60 in synovial fluid of RA vs OA patients. The dotted horizontal line represents the cut-off for positivity of the anti-HSP60 antibodies, which was determined as the mean±2SD of the 11 OA synovial fluid samples. **(b)** The bar graph shows the percentage of positive IgG, IgM and IgA anti-HSP60 antibodies in RA and OA patients. **(c)** The graph shows the percentage of synovial fluid samples in RA and OA containing no anti-HSP60 antibodies (negative), one (single positive), two (double positive) or three (triple positive) out of the three subclasses IgG, IgM and IgA anti-HSP60 antibodies.

Supplementary Figure S9. HSP60 analysis in pre- and post-tocilizumab treatment.

HSPD1 normalised synovial gene expression levels assessed at baseline in CDAI non-responders vs responders and DAS28-CRP moderate/non-responders vs responder patients to Tocilizumab (TOC) treatment.

Supplementary Figure S10. HSP60 analysis in pre- and post-rituximab treatment.

(a) *HSPD1* normalised gene expression levels assessed at baseline and 16-weeks following rituximab treatment in unpaired synovial biopsies (rituximab, n=72; tocilizumab, n=67). CDAI 50% improvement, DAS28 CRP and DAS28 ESR EULAR response were used to assess the clinical response. **(b)** Graphs showing correlation of IgG anti-HSP60 antibodies and CDAI 50% improvement, tender joints and DAS28 ESR EULAR clinical response at baseline before rituximab treatment.

Supplementary Figure S11. Anti-HSP60 antibodies analysis in synovial tissue and of pre- and post-rituximab treatment synovial biopsies.

(a) Anti-HSP60 IgG antibodies evaluation compared across histological pathotypes in established RA patients and **(b)** in patients stratified by histology into B-cell poor and B-cell rich. **(c)** Anti-HSP60 IgG antibodies assessed at baseline in CDAI non-responders vs responders and DAS28-ESR/CRP non-responder vs moderate/good responder patients to Rituximab treatment. **(d)** Anti-HSP60 IgG antibodies assessed at baseline and 16-weeks following rituximab treatment in CDAI/DAS28-ESR/CRP non-responder vs responder patients.

Supplementary Figure S12. Representative selection of areas of interest from multiple immunofluorescence staining of rheumatoid arthritis synovial tissue.

Representative immunofluorescence images stained for HSP60 (yellow). Nuclei were counterstained in DAPI (blue). Lymphoid aggregates are bordered in magenta, the peri-aggregate area in green, while overall section was delimited in red.
