# Supplementary figures and images for: Autoimmunity to stromal-derived autoantigens in rheumatoid ectopic germinal centers exacerbates arthritis and affects clinical response
Source: J Clin Invest. 2024 Apr 30;134(12):e169754. doi: 10.1172/JCI169754 (PMC11178537; doi:10.1172/JCI169754)

RA FLS extract vs 057/11.89.1.

2017/06/30  
LUCAS

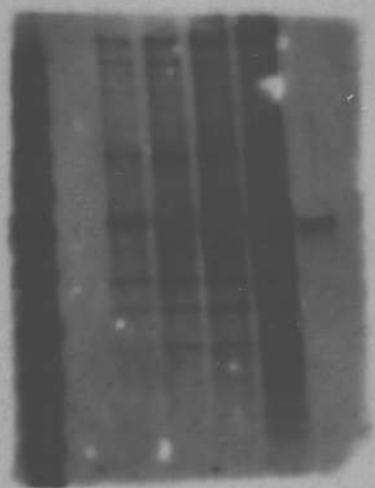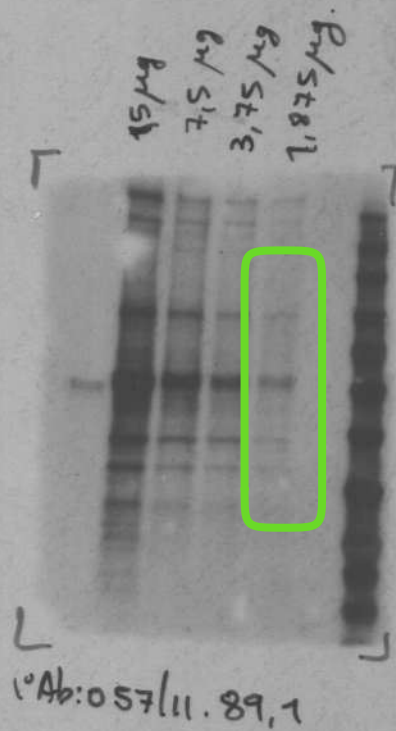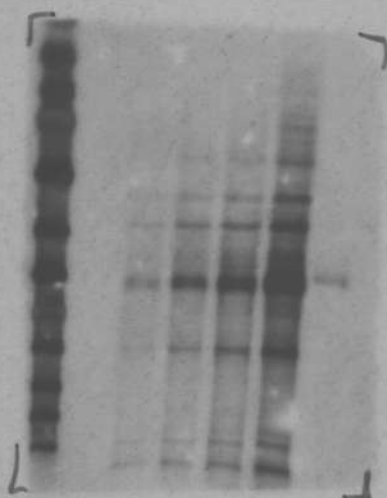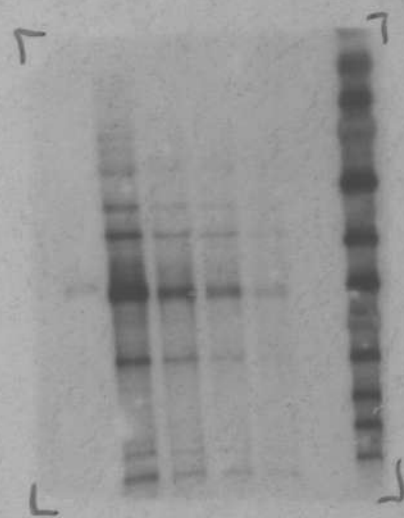

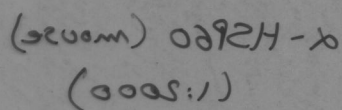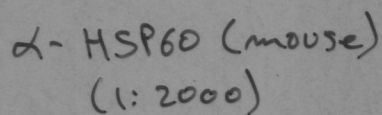

• 30  $\mu$ g Protein/lane.

### RA-FLS in starvation.

2018/10/24

2022/5/27

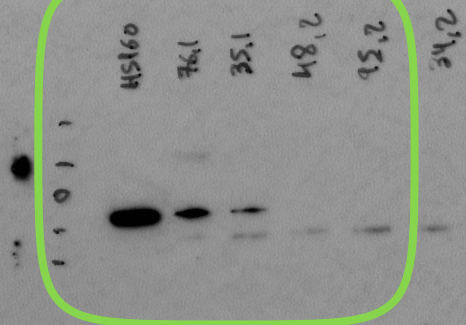

$\alpha$ -HSP60 (mouse)  
(1:2000)

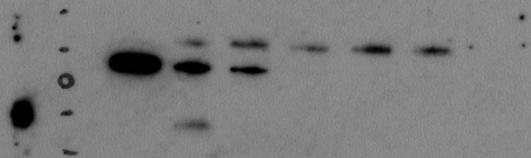

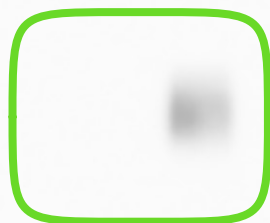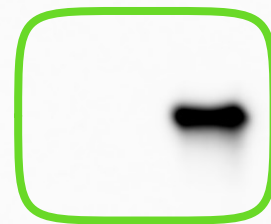

Supplement: Unedited blot and gel images [file jci-134-169754-s222.pdf]
